# Use of an asparaginyl endopeptidase for chemo-enzymatic peptide and protein labeling†
†Electronic supplementary information (ESI) available. See DOI: 10.1039/d0sc02023k


**DOI:** 10.1039/d0sc02023k

**Published:** 2020-05-12

**Authors:** T. M. Simon Tang, Davide Cardella, Alexander J. Lander, Xuefei Li, Jorge S. Escudero, Yu-Hsuan Tsai, Louis Y. P. Luk

**Affiliations:** a School of Chemistry , Cardiff University , Main Building, Park Place , Cardiff , CF10 3AT , UK . Email: lukly@cardiff.ac.uk

## Abstract

Asparaginyl endopeptidases (AEPs) are ideal for peptide and protein labeling. However, because of the reaction reversibility, a large excess of labels or backbone modified substrates are needed. In turn, simple and cheap reagents can be used to label N-terminal cysteine, but its availability inherently limits the potential applications. Aiming to address these issues, we have created a chemo-enzymatic labeling system that exploits the substrate promiscuity of AEP with the facile chemical reaction between N-terminal cysteine and 2-formyl phenylboronic acid (FPBA). In this approach, AEP is used to ligate polypeptides with a Asn–Cys–Leu recognition sequence with counterparts possessing an N-terminal Gly–Leu. Instead of being a labeling reagent, the commercially available FPBA serves as a scavenger converting the byproduct Cys–Leu into an inert thiazolidine derivative. This consequently drives the AEP labeling reaction forward to product formation with a lower ratio of label to protein substrate. By carefully screening the reaction conditions for optimal compatibility and minimal hydrolysis, conversion to the ligated product in the model reaction resulted in excellent yields. The versatility of this AEP-ligation/FPBA-coupling system was further demonstrated by site-specifically labeling the N- or C-termini of various proteins.

## Abstract 2

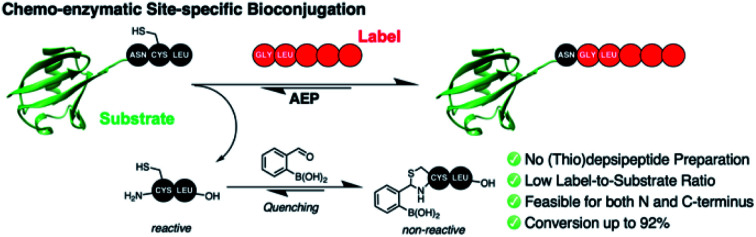
Asparaginyl endopeptidases (AEP) are ideal for peptide and protein labeling. Its pairing with a simple chemical reaction significantly lowers the amount of label needed for effective bioconjugation.

## Introduction

There has been a vast expansion in the toolkit of protein bioconjugation,^1^–^3^ drawing on expertise from both biological^4^–^6^ and organic^7^–^12^ chemistry. Protein-based approaches offer the ability to function efficiently under mild reaction conditions.^5^,^6^ Transferase,^13^–^15^ oxidoreductase,^16^–^18^ ligase,^19^,^20^ transpeptidase^2^,^4^,^21^–^27^ and (split-)intein^28^–^30^ have been applied for protein bioconjugation. Nevertheless, adapting these enzymes or proteins for synthetic applications, which deviate from their natural function, often results in technical issues. Whereas stability and solubility of intein-fused constructs are extremely case-dependent,^28^–^30^ reversible enzymatic reactions need to be suppressed by a large excess of label^24^,^31^ or unstable substrate that has inherent limitation to where bioconjugation takes place.^32^–^34^ In contrast, chemical approaches with commercially available reagents are simple to perform and have become standard practice.^4^,^8^–^10^ However, efficiency and selectivity of these reactions relies on the availability of specific residues, which varies greatly among proteins.^2^,^35^ These descriptions apply to the bioconjugation reaction mediated by the enzyme asparaginyl endopeptidase (AEP) and the chemical labeling of N-terminal cysteine by 2-formyl phenylboronic acid (FPBA). Here, we combine their strengths together, such that site-specific protein labeling can be achieved at the terminus of choice (N or C).

**Fig. 1:**
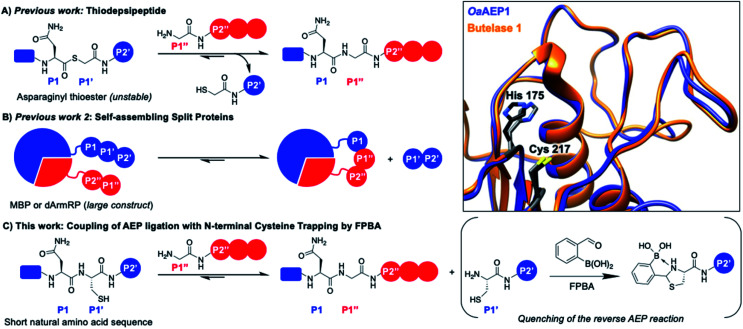
AEP catalyzes the hydrolysis of the amide bond between amino acids P1 (Asx) and P1′ followed by the ligation of the α-amine of P1′′. Various approaches have been reported to drive the reaction equilibrium towards product formation, including the use of (A) thiodepsipeptides^32^ and (B) self-assembling protein domains.^48^,^49^ (C) Here, a chemo-enzymatic approach that incorporates N-terminal cysteine trapping by formylphenyl boronic acid (FPBA) was reported. (Inset) Crystal structure overlay of the active sites of *Oa*AEP1 (orange, 5H0I) and butelase 1 (blue, 6DHI), with the catalytic diad of *Oa*AEP1 and butelase 1 highlighted in black and grey, respectively. Residues are numbered according to OaAEP1.

Many asparaginyl endopeptidases (AEPs) from plants possess transpeptidase function and present as ideal biocatalysts for protein labeling (*i.e.,* intermolecular ligation).^24^,^26^,^27^,^36^–^38^ AEP hydrolyzes the C-terminal amide bond of an internal asparagine or aspartate residue (P1) and subsequently mediate ligation to the N-terminus of an incoming nucleophile peptide (Fig. 1).^26^,^36^,^39^–^41^ Some important examples of AEP include butelase 1 and OaAEP1, and their excellent kinetic properties and relatively short recognition sequence (3 amino acids, P1–P1′–P2′) are the key advantages. Protein substrates including GFP, ubiquitin, ompA, DARPin, maltose binding proteins and nanobodies have previously been modified by AEPs, whereas unnatural amino acids, click handles, modified residues, polyethylene glycol, fluorophores, biotin and drug molecules have been used as labels.^24^,^26^,^31^,^32^,^42^–^45^ However, similar to other transpeptidases, bioconjugation by AEP requires a relatively large excess of labeling agents, with respect to the protein.^31^ This becomes a major hurdle for expensive or non-commercially available labels (*e.g.,* isotopic, radioactive and fluorescent labels).^46^–^49^

One valuable feature of AEPs is their relatively relaxed substrate specificity, which has been used to improve the enzyme-catalyzed bioconjugation reaction.^26^,^36^,^38^,^39^,^41^,^48^,^49^ Asparaginyl thiodepsipeptides have been used to develop irreversible butelase 1-mediated labeling (Fig. 1A). However, these alternative substrates have extremely short half-lives (*k*_1/2_ ≈ 45–75 min at pH 6.5) and effective labeling requires four to five equivalent excess of label, added in small portions.^32^,^50^ Moreover, labeling with the use thiodepsipeptide is limited to the N-terminus.^32^ Other solutions include the use of split proteins which have inherent affinity towards each other (Fig. 1B),^48^,^49^ but it requires the insertion of a large split-protein domain. Rehm *et al.* has illustrated that the reactivity difference between two recognition sequences at P2' (Asn–Gly–Leu *versus* Asn–Gly–Val) can be exploited to avoid hydrolysis catalyzed by OaAEP1, a homologue that carries significant sequence identity to butelase 1 (66%) (Fig. 1 inset).^24^ However, a large excess of labeling agents (20–120 equivalents, relative to the protein substrate) for intermolecular ligation was used. It has also been demonstrated Asn–Cys–Leu (P1–P1′–P2′) can be recognized for ligation by OaAEP1;^48^,^49^ such a reaction will generate byproduct that carries a N-terminal cysteine (Cys–Leu) whose reactivity can be exploited to develop an irreversible AEP-reaction and has yet to be explored.

The 1,2-aminothiol functionality of N-terminal cysteine contains two nucleophilic centers, and thus it can react with aldehydes to form thiazolidinones.^51^–^54^ 2-Formyl phenylboronic acid (FPBA) is one such electrophile that reacts with N-terminal cysteine with exquisite selectivity and efficiency.^53^,^54^ With biomolecular rate constant measured up to 10^5^ M^–1^ s^–1^, FPBA and its derivatives have been used for polypeptide labeling.^53^,^54^ Nevertheless, preparing proteins with a N-terminal cysteine is case-dependent and can be a challenging task.^30^,^55^–^59^ While successful examples have been reported, the N-terminal cysteine can readily react during gene expression (*e.g.* pyruvate) limiting the potential FPBA labeling reaction.^30^,^55^–^59^ Instead, this cheap and commercially available reagent can be used as a scavenger for AEP catalysis. We propose that the non-natural secondary amine motif formed between FPBA and N-terminal cysteine is unlikely to be a reactive substrate for AEP catalysis, and thus the byproduct of the enzymatic reaction Cys–Leu can be potentially trapped by the addition of FPBA. To this end, an irreversible AEP-labeling system can be achieved, such that bioconjugation can take place at the terminus of choice (N or C) with minimal modification using a lower ratio of label to protein (Fig. 1C).

Here, we develop a chemo-enzymatic protein labeling strategy, whereby the intermolecular ligation by AEP is coupled to a FPBA reaction that quenches the ligation byproduct. The P1–P1′–P2′ recognition sequence used for AEP labeling is Asn–Cys–Leu, and FPBA is added as a scavenger that reacts with the 1,2-aminothiol motif of Cys–Leu (Fig. 1C).^53^,^54^ Consequently, the AEP-mediated peptide ligation can be driven forward, whilst lowering the amount of labeling reagents used. By carefully tuning the amount of FPBA, pH, temperature and reaction time, the newly developed labeling system has minimal hydrolysis for the label, protein substrate and product. In our model peptide ligation reactions, this chemo-enzymatic approach proceeds in excellent yields (up to 95%). We have also proved our concept by labeling the N- or C-terminus of proteins of different sequences.

## Results and discussion

### Preparation of OaAEP1–C247A

The AEP variant derived from *Oldenlandia affinis Oa*AEP1–C247A was chosen due to the availability of its recombinant procedure, and this variant was also reported to have activity superior to that of the wild type and comparable to that of butelase 1 (under their respective optimized reaction conditions) (Fig. 1 inset).^24^,^39^ However, in the literature, a prodomain was included during gene expression, and thus enzyme activation under acidic condition was required.^24^,^26^,^37^ Although, in agreement with the literature,^26^,^37^ the activity of the activated enzyme can be mildly improved (∼15%, ESI Fig. S1†) after subjecting the activated OaAEP1–C247A to cation exchange chromatography, a lengthy preparation protocol is not desirable. With a view on practicality, we have simplified the enzyme preparation protocol by expressing the gene of the active enzyme fused with a ubiquitin and a hexahistidine tag. The enzyme yield is comparable to existing approaches (∼2 mg *vs.* 1.8 mg, see ESI† for more information),^26^ but the steps involved are reduced. Furthermore, this simplified enzyme construct showed comparable kinetic behaviors to the acid activated OaAEP1–C247A (for comparisons between the *k*_cat_ and *K*_M_ values, see Table S1†)^24^,^39^ and was shown to mediate protein bioconjugation *via* the chemo-enzymatic approach described below.

### Substrate specificity of OaAEP1–C247A

**Fig. 2:**
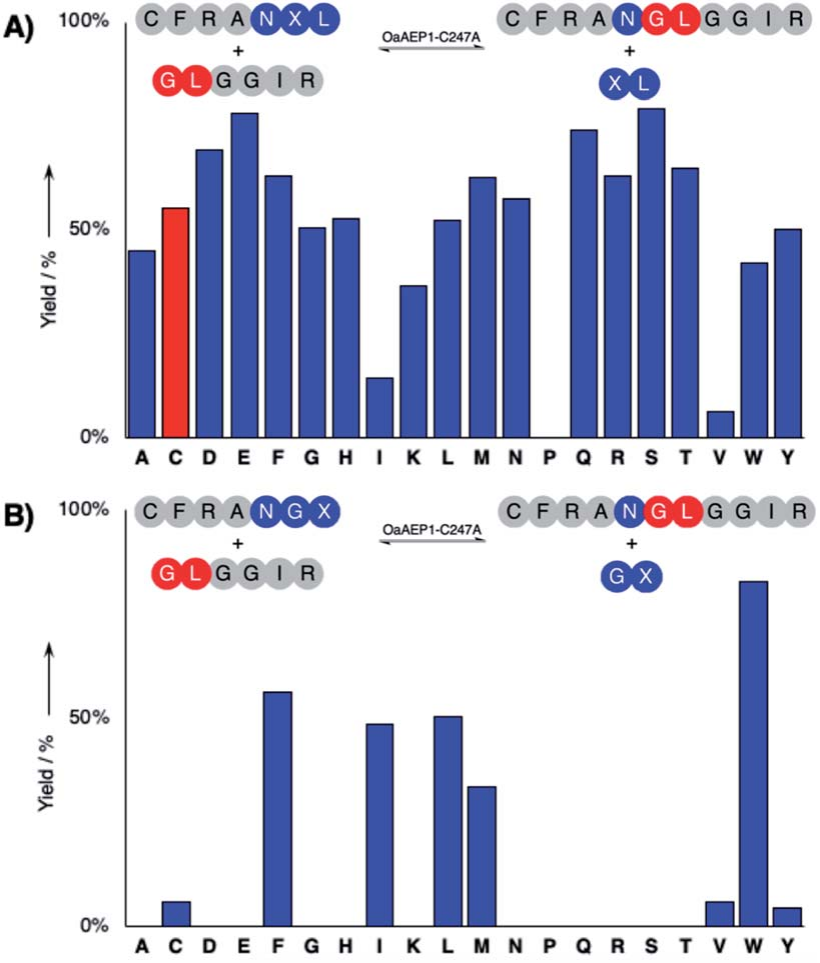
Hydrolysis profile for peptide ligation catalyzed by OaAEP1–C247A. (A) The ligation of CFRANXL to GLGGIR, with the investigated alternative recognition sequence (Asn–Cys–Leu) colored in red, and (B) the profile using CFRANGX, where X is any of the 20 amino acids.

The hydrolysis and nucleophile peptide profiles for peptide cyclization by OaAEP1 has been previously reported.^24^,^26^,^37^,^39^ To complement these reports, here we present the hydrolysis profile for intramolecular OaAEP1–C247A (Fig. 2). The model ligation reaction between CFRAN**X[combining low line]**L (where X at P1′ position is any of the 20 amino acids, 50 μM) and GLGGIR (250 μM, 5 equivalents) was performed at pH 5.0 with 0.1 μM of enzyme (1 : 500 enzyme to substrate ratio, Fig. 2A). Specificity at the P1′ position is rather relaxed; OaAEP1–C247A was able to hydrolyze the peptide bond between asparagine and all 20 amino acids except proline (Fig. 2A). Similar to the previous work that characterized the wild-type OaAEP1,^48^,^49^ Asn–Cys–Leu can also be recognized by the C247A variant, thus allowing us to develop the proposed coupling between AEP catalysis and cysteine/FPBA reaction (Fig. 1C). In contrast, specificity at the P2′ position is more restricted. When CFRANG**X[combining low line]** was used, the enzyme prefers large hydrophobic residues such as Phe, Ile, Leu, Met and Trp (Fig. 2B). Furthermore, echoing the report by Rehm *et al.*,^24^ our hydrolysis profile also illustrates that a Val residue at the P2′ position results in poor hydrolytic activity. The P2′ preference of OaAEP1–C247A also resembles the P2′′ acceptor profile of butelase 1, an AEP with significant sequence identity (66%) (Fig. 1; inset).^26^,^36^ Based on previous studies of OaAEP1,^24^,^26^,^37^,^39^ G and L at P1′′ and P2′′ represent one of the most effective combination for the nucleophile peptide. Nevertheless, other combinations of residues can also be used, as long as they do not interfere with the reactivity of FPBA (*e.g.,* avoid N-terminal cysteine) and are suitable for the specific AEP variant used.^39^

### Kinetic characterizations of OaAEP1–C247A and FPBA conjugation

The aim of this work is to quench the reactivity of the P1′–P2′ byproduct (Cys–Leu) generated from the AEP labeling reaction by including the electrophile FPBA (Fig. 1C). Consequently, the compatibility between AEP catalysis and cysteine/FPBA reaction was investigated. A model thiazolidine formation reaction between FPBA and a N-terminal cysteine peptide CFRANGL was monitored by a reported UV spectroscopic assay.^53^,^54^ Gratifyingly, the bioconjugation reaction took place at all examined pH's with rate constants ranging from 0.7 × 10^3^ M^–1^ s^–1^ at pH 5.0 to 6.0 × 10^3^ M^–1^ s^–1^ at pH 7.0 (Fig. S2†). For OaAEP1–C247A catalysis, the pH rate profile indicates that the catalytic turnover constant (*k*_cat_) can be as high as 7.5 s^–1^ at pH 5.0 and decreases to ∼1 s^–1^ at pH 7.5 at 20 °C. Since the Michaelis constant (*K*_M_) remains largely unchanged, the catalytic efficiency (*k*_cat_/*K*_M_) was found to be within a three-fold difference from pH 5.0 to 7.5 (2.8 to 0.9 × 10^4^ M^–1^ s^–1^) (Fig. S3†). In the other words, the two reactions are kinetically compatible at the examined pH range (5.0–7.0). Even at a low concentration (*e.g.,* μM to mM range), the rate of thiazolidine formation is compatible to the catalytic turnover for OaAEP1–C247A.

### Intermolecular peptide ligation mediated by OaAEP1–C247A

One consideration is that AEPs are able to catalyze hydrolysis of internal asparagine (and aspartate) residues using water as the nucleophile.^26^,^37^,^38^,^43^ Consequently, the label, substrate or product could be hydrolyzed to yield unwanted side-products. Using the intermolecular ligation reaction between the peptides LFRANCLK and GLGGIR (Table 1; eqn (1)) as a model, we have isolated several key variables that influence the extent of hydrolysis by OaAEP1–C247A.

**Table 1:** Model reaction catalyzed by OaAEP1–C247A in the absence and presence of 2-formyl phenylboronic acid (FPBA)^*a*^

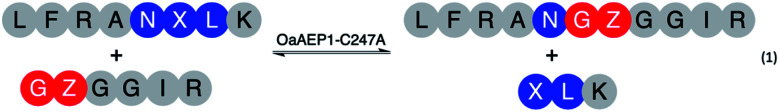							
Entry	pH	X	Z	GZGGIR^*b*^ (equiv.)	Time (h)	Conversion (%)	
						(–) FPBA	(+) FPBA
1	5.0^*c*^	Cys	Leu	1.2	3	53^*e*^	89^*e*^
2	5.2^*c*^				3	54^*f*^	95^*f*^
3	5.5^*d*^				3	49^*f*^	85^*f*^
4					4	48^*f*^	90^*f*^
5	5.7^*d*^				3	51^*g*^	78^*g*^
6					4	50^*g*^	94^*g*^
7	5.7^*d*^	Ala			3	67^*g*^	61^*g*^
8					4	70^*g*^	67^*g*^
9	5.7^*d*^	Cys	Val	1.0	4	24^*f*^	45^*f*^
10				1.5		33^*f*^	55^*f*^
11				2.0		32^*f*^	66^*f*^
12				5.0		65^*g*^	>95^*g*^
13				10.0		>95^*g*^	>95^*g*^
14				20.0		>95^*g*^	>95^*g*^

^*a*^All reactions were carried out in triplicate with OaAEP1–C247A (0.3 μM), LFRANXLK (300 μM) and GZGGIR (300–6000 μM) at 20 °C. OaAEP1–C247A prepared from acid activation of the zymogen and from the simplified construct were used.

^*b*^Equivalents of labeling peptide used relative to the peptide substrate, LFRANXLK.

^*c*^50 mM NaOAc buffer with 50 mM NaCl, 1 mM EDTA, 0.5 mM TCEP.

^*d*^50 mM MES buffer with 50 mM NaCl, 1 mM EDTA, 0.5 mM TCEP.

^*e*^10–15%.

^*f*^5–10%.

^*g*^<5% of the undesired hydrolysis product observed.

The pH of the reaction was kept at ≥5.0 to minimize the undesired hydrolytic reaction (Table 1; entry 1–6. Table S2;† entry 42–43, 56–57, 62–63, 72–73 for reactions tested at pH 4.5–5.7); similar observations were made in the studies of AEP from other plant species.^36^,^40^ Hydrolysis was found to be further minimized when the temperature was kept at ≤20 °C (Table S2;† entry 46–53 and 92–96 for reactions tested at 20 and 37 °C). Also, reaction time needed to be carefully screened in order to strike a balance between hydrolysis and product formation (Table S2;† entry 58–59). While AEP can hydrolyze peptide upon prolonged incubation,^36^,^40^ FPBA was also found to slow the process of peptide ligation. This is likely because of reversible interactions with either the enzyme or the nucleophilic peptide (*e.g.,* forming iminium ion; Table 1; entry 3–6 and entry 80–85 in Table S2†). Based on the +100 polypeptide reactions tested here, the reaction time was kept in between 2 and 18 h, with a mode average of 4 h.^24^ Lastly, a relatively small excess of the nucleophilic peptide (1.2–2 equivalents) was used to further diminish hydrolysis (Tables 1 and 2 and entry 30–32 in Table S1†).

**Table 2:** C-terminus labeling of eGFP with biotinylated peptide using OaAEP1–C247A in the presence of 2-formyl phenylboronic acid (FPBA)

Entry	GLGGZ^*a*^ (equiv.)	Yield (%)	Hydrolysis (%)
1	1.0	63	4
2	1.2	66	1
3	1.5	75	2
4	2.0	86	0

^*a*^Equivalents of labeling peptide used relative to the protein substrate eGFP. It should be noted that an Asp-to-Ala mutation was needed to avoid undesired side-product (Table S3).

Under the optimized conditions, 94% of the ligated product LFRANGLGGIR was obtained in 4 h at pH 5.7 using 1.2 equivalent of the nucleophilic peptide, when FPBA was included in the system (Table 1; entry 6). In contrast, when FPBA was excluded, product conversion was stalled at ∼50% (Table 1; entry 1–6). For the cysteine-free peptide LFRANALK, no significant difference in ligation yield was observed by adding FPBA (Table 1; entry 7–8). Also, the enzymatic activity difference between the P1–P1′–P2′ recognition sequences Asn–Cys–Leu and Asn–Gly–Leu was only about 10% (Fig. 2A). Together, these observations indicate that the increase of reaction yield (>40%, Table 1) is caused by the coupling between the FPBA reaction and AEP ligation.

Recently, it has been demonstrated that hydrolysis can be avoided by using polypeptides that carry Gly–Val as the P1′′–P2′′ nucleophile.^24^ To examine if this concept can be applied to the chemo-enzymatic strategy described here, an alternative peptide GVGGIR was tested in the model ligation reaction (Table 1; entries 9–14).^24^ Independent of FPBA addition, excellent conversion (>95%) was observed using 10–20 equivalents of peptides. However, when only 1–5 equivalents of GVGGIR were used, conversion was found to be ∼1.5 to 2-fold higher in the reactions that included FPBA. Nevertheless, product conversion was notably lower when compared to those where GLGGIR were used, thus suggesting that Gly–Val nucleophile is less suitable for the chemo-enzymatic approach. Consequently, nucleophilic peptides with Gly–Leu at the N-terminus were used for protein bioconjugation.

### OaAEP1–C247A mediated protein bioconjugation

**Fig. 3:**
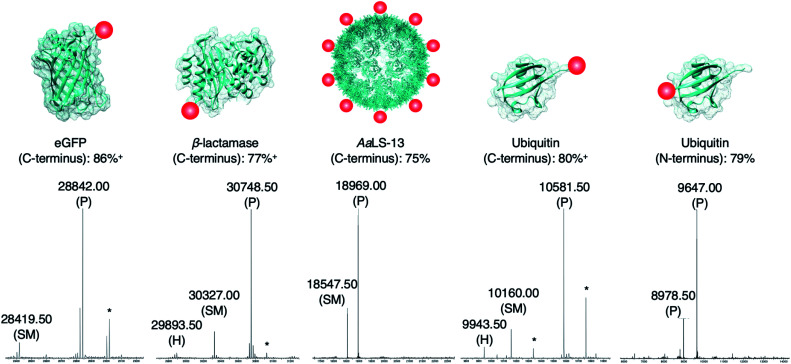
Protein modification. Proteins (100 μM) modified with a short linker and the recognition sequence (Asn–Cys–Leu) for OaAEP1 were incubated with biotin labeled peptide (200 μM), OaAEP1-C247A (0.25 μM) and FPBA (200 μM). The reactions were quenched with 1 M HCl then analyzed by UPLC-MS. SM refers to starting material and P to product. (*) Denotes peaks correlating to α-N-gluconoylation of recombinant protein starting material and products (+178 Da).^61^,^62^ (+) Denotes conversion averaged from repeated experiments, associated errors are reported in Fig. S5, S6 and S8.† Asp-to-Ala mutation was introduced to eGFP in order to avoid undesired hydrolytic reaction (Table S3†). The corresponding chromatograms and full mass spectra of the UPLC-MS analysis are reported in Fig. S5–S9.† Other than the species reported above, there is no evidence of hydrolyzed peptides or formation of any other byproducts. Quenching by the addition of 1 M HCl was used solely for the purpose of obtaining a precise reaction time during optimization.

The newly developed AEP-ligation/FPBA-coupling approach was employed for site-specific C-terminal labeling. A linker containing Asn–Cys–Leu was added to the C-terminus of enhanced green fluorescent protein (eGFP), and ligation reactions with the biotinylated peptide GLGGZ (where Z is biotinylated lysine) were performed. Estimated by LC-MS analysis,^10^,^24^,^60^ the yield of the C-terminally modified protein increased up to 1.5-fold when FPBA is included (Table S3†), and up to 92% of the C-terminally modified protein could be achieved using two equivalents of the biotin label (Table 2 and Fig. 3). Similar findings were observed in the labeling of other monomeric and multimeric proteins. 85% of β-lactamase was modified at the C-terminus under the same reaction condition (Fig. 3). As demonstrated here and in previous works,^27^,^37^ AEP functions from pH ∼5.0–7.5, but some proteins may become unstable even under mildly acidic environments. Hence, the presented labeling system was also tested with the engineered lumazine synthase AaLS-13, a macromolecular complex composed of 360 protein subunits which is prone to precipitation at pH below 7.0.^63^–^65^ Up to 75% of the AaLS-13 subunits were found to be labeled under neutral pH condition (Fig. 3). While it is of little doubt that full conversion can be achieved by using a slightly higher equivalent of labeling peptide (Table 2), reactions performed here were capped at two equivalents to demonstrate the effectiveness of this approach at a relatively low label-to-protein ratio. In a similar fashion, ubiquitin bearing a C-terminal Asn–Cys–Leu sequence could be labeled with the biotinylated peptide GLGGZ (Fig. 3). These findings illustrate that the chemo-enzymatic approach is suitable for a range of proteins with different sequence and biophysical properties, whilst lowering the ratio of label to protein substrate needed, and is complementary to the existing site-specific C-terminal modification technologies.^12^,^24^,^32^,^42^–^44^,^60^,^66^,^67^

Since only natural amino acids are needed for recognition, the chemo-enzymatic method was also applied for site-specific N-terminus labeling. Ubiquitin containing an extra Gly–Leu sequence at the N-terminus was recombinantly prepared as the substrate, and biotin-labeled peptide bearing the recognition sequence Asn–Cys–Leu at the C-terminus (*i.e.*, biotin-ATRNCL) was synthesized for labeling (see ESI†). 79% of ubiquitin was labeled at the N-terminus using two equivalents of the biotinylated label when FPBA was included (Fig. 3). This finding is complementary to the previous work in which thiodepsipeptide was used,^32^ but issue surrounding the stability of label was not observed here. It should be noted that all the proteins used here contained other internal asparagine and aspartate residues. However, they are neither hydrolyzed nor modified with only one exception. An Asp235Ala mutation at the solvent-exposed internal site of eGFP (11 residues from the C-terminus) was needed (Fig. S5 and Table S4†).^68^ In the other words, accessibility plays a critical role in AEP-based modification, dictating both the reaction yield and side reactions.

## Conclusions

The presented work combines the advantages of chemical and enzymatic labeling, creating a bioconjugation system with features that could not be achieved by either method alone. Transpeptidases are appealing tools for bioconjugation,^5^,^6^,^13^,^14^,^16^,^21^,^26^,^39^,^42^ but their reactions are reversible, and thus a large ratio of labeling agents to protein substrate is needed to achieve high conversion.^21^,^23^,^32^,^53^,^54^,^60^,^69^ While the use of backbone-modified labels such as (thio)depsipeptides has improved yields of reactions catalyzed by sortase,^33^,^34^ subtiligase,^70^,^71^ trypsiligase^22^,^25^ and AEPs,^32^ stability of these alternative labels varies significantly and can be difficult to prepare.^50^ Furthermore, the backbone-modified approach is largely limited to N-terminal labeling.^32^,^33^,^70^ On the other hand, FPBA is a commercially available reagent that offers a fast, selective and simple method for modification,^4^,^53^,^54^ but preparation of proteins with a free N-terminal cysteine is not universally trivial, as it is prone to side reaction (oxidation and thiazolidinone formation).^30^,^55^–^59^ However, when the AEP catalysis and FPBA bioconjugation are combined together, a system that enables both N- and C-terminal ligation with the use of stable labeling agents is developed.^32^

Provided that the AEP-ligation/FPBA-coupling method lowers the ratio of label to protein substrate, it is particularly applicable when expensive or non-commercially available labels are used.^46^–^49^ While some of the proteins labeled here, including eGFP, beta-lactamase and AaLS13, contain cysteine residues (in oxidized or reduced form), it should be noted that extension of this approach might require preliminary investigations, as addition of cysteine residue could potentially destabilize some proteins,^72^,^73^ particularly those that contains significant number of disulfide bonds (*e.g.,* anti- and nano-bodies).^72^,^73^ Since the activity of OaAEP1 is limited to neutral or acidic conditions,^37^ studies towards the use of FPBA to enhance other enzymatic labeling strategies may be worthwhile. The development in this technology complements the use of existing transpeptidases such as sortase, as the differences in substrate specificity may be fully exploited in combination to develop orthogonal ligation strategies.^23^,^24^,^44^ In summary, the pairing of enzymatic transpeptidation with well-established chemical reactions offers a versatile and efficient approach to the preparation of tailored protein constructs.

## Conflicts of interest

The authors declare no conflicts of interest.

## Supplementary Material

Supplementary information
