# Supplementary material for: Use of an asparaginyl endopeptidase for chemo-enzymatic peptide and protein labeling
Source: Chem Sci. 2020 May 12;11(23):5881–8. doi: 10.1039/d0sc02023k (PMC7441500; doi:10.1039/d0sc02023k)
Supplement: Supplementary file 1 [file SC-011-D0SC02023K-s001.pdf]

Electronic Supplementary Material (ESI) for Chemical Science.
This journal is © The Royal Society of Chemistry 2020

Supplementary Information

Use of an Asparaginyl Endopeptidase for Chemo-enzymatic Peptide and Protein Labeling

T. M. Simon Tang[a], Davide Cardella[a], Alexander J. Lander[a], Xuefei Li[a], Jorge S. Escudero[a], Yu-Hsuan Tsai[a], Louis Y. P. Luk*[a]

Experimental Procedures

Chemistry

Materials

Low Loading Rink-Amide resin (Fluorochem) was used for solid phase peptide synthesis. Deionized water was obtained from an Elga PURELAB Option system (15 MΩ·cm). Reagents obtained from commercial suppliers were used without further purification unless otherwise stated. Fmoc-amino acids(PG)-COOH, N, N, N′, N′-tetramethyl-O-(1H-benzotriazol-1-yl) uronium hexafluorophosphate (HBTU) and hydroxybenzotriazole (HOBT), triisopropylsilane (TIS) and 3,6-dioxa-1,8-octane-dithiol (DODT) were purchased from Cambridge Reagents. N, N-Diisopropylethylamine, piperidine and trifluoroacetic acid were purchased from Fluorochem. N, N-dimethylformamide (DMF) was purchased from Cambridge Reagents and dichloromethane (DCM) from Fisher Scientific. For HPLC mobile phase, HPLC grade acetonitrile (ACN) from Fisher Scientific and trifluoroacetic acid from Fluorochem were used.

LCMS method

Liquid chromatography–mass spectrometry (LCMS) was performed on a 1260 Infinity II from Agilent Technologies employing an Eclipse plus C18 column, 3.5 µm particle size, 95 Å pore size, 4.6 mm × 100 mm. If not stated otherwise the following method was employed: 40 °C, flow rate = 0.5 mL·min^{-1}, H_2O:ACN (0.1% HCO_2H) 100:0, 5 min, 95:5 gradient to 3:97 over 18 min, 1 min hold, gradient to 97:3 over 1 min.

Preparative HPLC

Reverse phase high performance liquid chromatography (RP-HPLC) was performed on an Agilent 1260 Infinity HPLC using a Phenomex Gemini C18 column, 10 µm particle size, 110 Å pore size, 10.0 mm × 250 mm. Flow rate = 4.0 mL·min^{-1}, H_2O:ACN (0.1% HCO_2H) 97:3, 5 min, 97:3 gradient to 72.5:27.5 over 35 min, 0.1 min hold, gradient to 5:95 over 4.9 min.

Solid phase peptide synthesis

All peptides were prepared by Fmoc-solid-phase peptide synthesis on low loading rink-amide resin with Fmoc amino acids (2 equiv.), HOBT(4 equiv.), HBTU (4 equiv.) and DIPEA (8 equiv.). Each coupling reaction was performed once. Deprotection of side chains and cleavage from the solid support was performed with TFA/H_2O/TIS/DODT (92.5:2.5:2.5:2.5) at 25 °C for 3 h. The mixture was then filtered off through glass wool and concentrated under N_2 flow. The crude peptide was triturated with cold Et_2O then centrifuged at 4,000 g for 10 minutes. The supernatant was discarded, the pellet was dissolved with H_2O/ACN (1:1) containing 0.1% TFA, filtered then purified by HPLC. The identity of the purified peptides were confirmed by LCMS.

Biochemistry

LCMS method

LCMS was performed on infinity II HPLC system coupled to ESI single quadrapole mass spectrometer (Agilent), using an eclipse plus C18 (100 x 4.6 mm, Agilent) with a linear gradient from 5% to 35% acetonitrile (0.1% formic acid) over 15 min.

Analytical HPLC method

HPLC was performed on infinity II HPLC system (Agilent), using gemini C18 (250 x 10 mm, 110 Å Phenomenex) with a linear gradient from 5% to 30% acetonitrile (0.1% formic acid) over 15 min.

Protein UPLC-MS

Liquid chromatography-mass spectrometry (LC-MS) was performed on a Waters Synapt G2-Si quadrupole time-of-flight mass spectrometer coupled to a Waters Acquity H-Class ultraperformance liquid chromatography (UPLC) system. The column was an Acquity UPLC protein BEH C4 (300 Å, 1.7 µm by 2.1 mm by 100 mm) operated in reverse phase and held at 60 °C. The gradient employed was 95% A to 35% A over 50 min, where A is water with 0.1% HCO_2H and B is acetonitrile with 0.1% HCO_2H. Spectra were collected in positive ionization mode and analyzed using Waters MassLynx software version 4.1. Deconvolution of protein charged states was obtained using the maximum entropy 1 processing software.

Materials

Custom oligonucleotides were purchased from Merck Sigma Aldrich. Buffers, nucleotide triphosphate, and PrimeSTAR® HS DNA polymerase were purchased from Takara. All restriction enzymes were purchased from ThermoFisher Scientific. NEBuilder® HiFi DNA assembly master mix was purchased from New England Biolabs.

A gene with codons optimized for expression in *E. coli*, encoding a protein composed of a N-terminal hexa-His tag, the 76 amino-acid residues of human ubiquitin and residues 24 – 474 of OaAEP1 (C247A) was inserted into the coding region (NcoI-NdeI) of the pET-28b (+) vector (Genscript).

Molecular Cloning

OaAEP1-C247A core domain. The plasmid pET28b-His-Ub-OaAEP1 was used for preparing N-terminal His_6 tagged OaAEP1-C247A by IPTG induction. It was constructed by subcloning the gene encoding human ubiquitin using primers ST28FV and ST29R. The PCR product was digested by DpnI (ThermoFisher) then transformed into DH5α *Escherichia coli*. Sequences of all plasmids were confirmed by DNA Sanger sequencing performed by Eurofins Genomics.

ST28FV

AAGCTTGCGGCCGCACTCGAGGAGATCCGGCTGCTAACAAAGCC

ST29R:

CTCGAGTGCGGCCGCAAGCTTTTAGTCGTTCGCCGGGTTGC

Ubiquitin. The plasmid pET28b-His-TEV-linker-Ub was used for the IPTG-inducible production of the N-terminal His_6 tagged ubiquitin and was constructed by subcloning the gene encoding human ubiquitin into pET28b using primers ST21F and ST21R via Gibson assembly. For ubiquitin bearing the C-terminal recognition sequence (NCL), the gene encoding for ubiquitin was amplified from ST21 using primers ST23F and ST23R, then subcloned into a pET28b derived vector by Gibson assembly to generate the plasmid pET28b-His-Ub-NCL. Finally, a Gly to Pro site directed mutagenesis in the C-terminal linker region was carried out using primers ST23F G76P and ST23R G76P to prevent undesired cleavage during

recombinant expression. Sequences of all plasmids were confirmed by DNA Sanger sequencing performed by Eurofins Genomics.

ST21F: (His_TEV_GL_linker_Ub)
GGTCTGAGCGGTAGCGGTAGCCAGATCTTCGTTAAAACCCTGACCGGC
ST21R:
CTGGCTACCGCTACCGCTCAGACCCTGGAAGTACAGGTTCTCACC
ST23F: (His_Ub_NCL)
TGCACCACCACCACCACCACCAGATCTTCGTTAAAACCCTGACCGGC
ST23R:
AAGCAATTACCGCTACCACCGGATCCGCCACCACGCAGACGCAG
ST23F G76P: (His_Ub_NCL)
GTGGTGGCCCGTCCGGTGGTAGCGGTAATTGC
ST23R G76P:
CACCGGACGGGCCACCACGCAGACGC

Green fluorescent protein. The plasmid pET28b-eGFP-NCL was used for the IPTG-inducible production of the N-terminal His_6 tagged enhanced green fluorescent protein (eGFP) and was constructed by subcloning the gene encoding eGFP using primers ST9FN and ST9RN, into a pET28b derived vector by Gibson assembly. For eGFP bearing the N-terminal His_6 tag followed by TEV and *Oa*AEP1 recognition sequences, the gene encoding for His_6-TEV-eGFP was amplified from ST9N using primers ST22F and ST22R, then subcloned into a pET28b derived vector by Gibson assembly to generate the plasmid pET28b-His-TEV-eGFP. Finally, Asp to Ala site directed mutagenesis were carried out on both ST9N and ST22 using primers GFP D235A For and GFP D235A Rev to prevent undesired cleavage during recombinant expression. Sequences of all plasmids were confirmed by DNA Sanger sequencing performed by Eurofins Genomics.
ST9FN (His_eGFP_NCL):
TTGTTTAACTTTAAGAAGGAGATATACATATGCACCACCACC
ST9RN:
ACGGAGCTCGAATTCGGATCTTATAAGCAATTACCGCTACCACCCTTGTACAGCTCGTC
CATGCCGAG
ST22F: (His_TEV_GL_linker_eGFP)
GCGGTAGCGGTAGCCATATGGTGAGCAAGGGCGAGGAG
ST22R:
TGTCGACGGAGCTCGAATTCTTAGGATCCCTTGTACAGCTCGTCCATGCCG
GFP D235A For: (D235A SDM)
CGGCATGGCCGAGCTGTACAAGGG
GFP D235A Rev:
CAGCTCGGCCATGCCGAGAGTGATC

Beta lactamase. The plasmid pET28b-His-TEV-BlaC (ST24) was used for the IPTG-inducible production of the N-terminal His_6 tagged beta-lactamase (BlaC) and was constructed by subcloning the gene encoding BlaC using primers ST24F and ST24R, into a pET28b derived vector by Gibson assembly. For BlaC bearing the N-terminal His_6 tag and a C-terminal

*Oa*AEP1 recognition sequence (NCL), the gene encoding for BlaC was amplified using primers ST25F and ST25R, then subcloned into a pET28b derived vector by Gibson assembly to generate the plasmid pET28b-His-BlaC-NCL (ST25). Sequences of all plasmids were confirmed by DNA Sanger sequencing performed by Eurofins Genomics.

ST24F (His_TEV_BlaC):
TGAGCGGTAGCGGTAGCCATATGGGTGCAGATCTGGCAG
ST24R:
GACGGAGCTCGAATTCTTAGGATCCTGCCAGAACACCG
ST25F: (His_BlaC_NCL)
TGCACCACCACCACCACCACATGGGTGCAGATCTGGCAG
ST25R:
AAGCAATTACCGCTACCACCGGATCCTGCCAGAACACCGGCAAC

AaLS 13. The plasmid pET28b-His-TEV-AaLS13 (ST26) was used for the IPTG-inducible production of the N-terminal His_6 tagged AaLS13 and was constructed by subcloning the gene encoding AaLS13 using primers ST26F and ST26R, into a pET28b derived vector by Gibson assembly. For AaLS13 bearing the N-terminal His_6 tag and a C-terminal *Oa*AEP1 recognition sequence (NCL), the gene encoding for AaLS13 was amplified using primers ST27F and ST27R, then subcloned into a pET28b derived vector by Gibson assembly to generate the plasmid pET28b-His-AaLS13-NCL (ST27). Sequences of all plasmids were confirmed by DNA Sanger sequencing performed by Eurofins Genomics.
ST26F (His_TEV_AaLS13):
TGAGCGGTAGCGGTAGCCATATGGAAATCTACGAAGGTAAACTAACTGCTGAAG
ST26R:
GACGGAGCTCGAATTCTTAGGATCCCTCGAGTCGGAGAGACTTGAATAAGTTTGC
ST27F: (His_AaLS13_NCL)
TGCACCACCACCACCACCACATGGAAATCTACGAAGGTAAACTAACTGCTGAAG
ST27R:
AAGCAATTACCGCTACCACCGGATCCCTCGAGTCGGAGAGACTTGAATAAGTTTGC

Protein expression and purification

OaAEP1-C247A core domain. Recombinant expression of ST29 was performed in *E. coli*, BL21(DE3). The cells were grown at 37 °C in LB medium until the OD_{600} reached 0.6, at which point protein production was induced by adding IPTG (0.2 mM). After culturing at 16 °C for 18 h, cells were harvested by centrifugation at 5000 g and 4 °C for 20 min. Cell pellets were stored at -20 °C until purification.
The cell pellet from a 1 L culture was suspended in 20 mL of lysis buffer containing 50 mM sodium phosphate (pH 8.0) 300 mM NaCl, 0.1 mg/mL lysozyme, DNase I, RNase A and PMSF. After lysis by sonication and clearance by centrifugation (27000g, 4 °C, 15 min), the supernatant was loaded onto 4 mL of Ni-NTA resin (Bio-Rad) in a gravity flow column (Bio-Rad). The column was washed three times with lysis buffer containing 10 mM imidazole (40 mL each), and the remaining bound protein was eluted with 20 mL of lysis buffer containing 300 mM imidazole. Ni-NTA elution was concentrated using an Amicon Ultra-15 centrifugal filter unit (10 kDa MWCO) (Merck Millipore), then further purified by size exclusion

chromatography (superdex 75, GE Healthcare) that had been pre-equilibrated in 50 mM MES (pH 6.0), 50 mM NaCl, 1 mM EDTA and 0.5 mM TCEP. Fractions containing desired protein were combined, concentrated using an Amicon Ultra-15 centrifugal filter unit (10 kDa MWCO) (Merck Millipore) then stored at -80 °C with 5% (v/v) glycerol.

Ubiquitin. Recombinant expression of Ubiquitin proteins was performed in *E. coli*, BL21(DE3). The cells were grown at 37 °C in LB medium until the OD_{600} reached 0.6, at which point protein production was induced by adding IPTG (0.1 mM). After culturing at 20 °C for 18 h, cells were harvested by centrifugation at 5000 g and 4 °C for 20 min. Cell pellets were stored at -20 °C until purification.
The cell pellet from a 1 L culture was suspended in 20 mL of lysis buffer containing 50 mM sodium phosphate (pH 8.0) 300 mM NaCl, 0.1 mg/mL lysozyme, DNase I, RNase A and PMSF. After lysis by sonication, and clearance by centrifugation (27000g, 4 °C, 15 min), the supernatant was loaded onto 4 mL of Ni-NTA resin (Bio-Rad) in a gravity flow column (Bio-Rad). The column was washed three times with lysis buffer containing 10 mM imidazole (40 mL each), the remaining bound protein was eluted with 20 mL of lysis buffer containing 300 mM imidazole. The eluant from the Ni-NTA chromatography was concentrated using an Amicon Ultra-15 centrifugal filter unit (3 kDa MWCO) (Merck Millipore), then further purified by size exclusion chromatography (superdex 75, GE Healthcare) that had been pre-equilibrated in 50 mM MES (pH 6.0), 150 mM NaCl, 1 mM EDTA, 0.5 mM TCEP. Fractions containing desired protein were combined, concentrated using an Amicon Ultra-15 centrifugal filter unit (3 kDa MWCO) (Merck Millipore) then stored at -80 °C. For protein constructs bearing TEV cleavage site (ST21), Ni-NTA elution fractions containing the desired protein were combined, buffer exchanged into 50 mM sodium phophate (pH 7.0), 150 mM NaCl, 1mM EDTA, 1 mM TCEP-HCl using an Amicon Ultra-15 centrifugal filter unit (3 kDa MWCO) (Merck Millipore). The protein was concentrated to around 5 mg/mL, then treated with TEV protease (1 mol%) overnight at 20 °C. The processed ubiquitin was isolated from the TEV cleavage reaction mixture by passing over 4 mL of Ni-NTA resin (Bio-Rad) in a gravity flow column (Bio-Rad). The flow through containing the desired protein was concentrated using an Amicon Ultra-15 centrifugal filter unit (3 kDa MWCO) (Merck Millipore), then purified by size exclusion chromatography as described above. Fractions containing desired protein were combined, concentrated using an Amicon Ultra-15 centrifugal filter unit (3 kDa MWCO) (Merck Millipore) then stored at -80 °C.

Enhanced green fluorescent protein (eGFP). Recombinant expression of eGFP was performed in BL21(DE3) *E. coli*. The cells were grown at 37 °C in LB medium until the OD_{600} reached 0.6, at which point protein production was induced by adding IPTG (0.1 mM). After culturing at 20 °C for 18 h, cells were harvested by centrifugation at 5000 g and 4 °C for 20 min. Cell pellets were stored at -20 °C until purification.
The cell pellet from a 1 L culture was suspended in a lysis buffer containing 50 mM sodium phosphate (pH 8.0) 300 mM NaCl, 0.1 mg/mL lysozyme, DNase I, RNase A and PMSF. After lysis by sonication, and clearance by centrifugation (27000g, 4 °C, 15 min), the supernatant was loaded onto 4 mL of Ni-NTA resin (Bio-Rad) in a gravity flow column (Bio-Rad). The column was washed three times with lysis buffer containing 10 mM imidazole (80 mL each), the remaining bound protein was eluted with lysis buffer containing 300 mM imidazole. Ni-NTA

elution fractions containing the desired protein were combined, buffer exchanged into 50 mM MES (pH 6.0) 150 mM NaCl, 1 mM EDTA, 1 mM TCEP-HCl using an Amicon Ultra-15 centrifugal filter unit (10 kDa MWCO) (Merck Millipore). The concentrated protein was then purified through a SEC column (superdex 75, GE Healthcare) that had been pre-equilibrated in 50 mM MES (pH 6.0) 150 mM NaCl, 1 mM EDTA, 1 mM TCEP-HCl. Fractions containing desired protein were combined, concentrated using an Amicon Ultra-15 centrifugal filter unit (10 kDa MWCO) (Merck Millipore) then stored at -80 °C.

***Mycobacterium tuberculosis* β-lactamase (BlaC).** Recombinant expression of BlaC was performed in *E. coli*, BL21(DE3). The cells were grown at 37 °C in LB medium until the OD_{600} reached 0.6, at which point protein production was induced by adding IPTG (0.1 mM). After culturing at 20 °C for 18 h, cells were harvested by centrifugation at 5000 g and 4 °C for 20 min. Cell pellets were stored at -20 °C until purification.
The cell pellet from a 1 L culture was suspended in a lysis buffer containing 50 mM sodium phosphate (pH 8.0) 300 mM NaCl, 0.1 mg/mL lysozyme, DNase I, RNase A and PMSF. After lysis by sonication, and clearance by centrifugation (27000g, 4 °C, 15 min), the supernatant was loaded onto 4 mL of Ni-NTA resin (Bio-Rad) in a gravity flow column (Bio-Rad). The column was washed three times with lysis buffer containing 10 mM imidazole (80 mL each), the remaining bound protein was eluted with lysis buffer containing 300 mM imidazole. Ni-NTA elution fractions containing the desired protein were combined, buffer exchanged into 50 mM MES (pH 6.0) 150 mM NaCl, 1 mM EDTA, 1 mM TCEP-HCl using an Amicon Ultra-15 centrifugal filter unit (10 kDa MWCO) (Merck Millipore). The concentrated protein was then purified through a SEC column (superdex 75, GE Healthcare) that had been pre-equilibrated in 50 mM MES (pH 6.0) 150 mM NaCl, 1 mM EDTA, 1 mM TCEP-HCl. Fractions containing desired protein were combined, concentrated using an Amicon Ultra-15 centrifugal filter unit (10 kDa MWCO) (Merck Millipore) then stored at -80 °C.

***Aquifex aeolicus* lumazine synthase-13 (*Aa*LS13).** Recombinant expression and purification of *Aa*LS13 was performed according to protocols reported previously.[1] The gene encoding for His_AaLS13_NCL was transformed into *E. coli* BL21(DE3). The cells were grown at 37 °C in LB medium until the OD_{600} reached 0.6, at which point protein production was induced by adding IPTG (0.1 mM). After culturing at 25 °C for 18 h, cells were harvested by centrifugation at 5000 g and 4 °C for 20 min. Cell pellets were stored at -20 °C until purification.
The cell pellet from a 1 L culture was suspended in a lysis buffer containing 50 mM sodium phosphate (pH 8.0) 300 mM NaCl, 0.1 mg/mL lysozyme, DNase I, RNase A and PMSF, then incubated at room temperature for 1 hour. After lysis by sonication, and clearance by centrifugation (27000g, 25 °C, 15 min), the supernatant was loaded onto 4 mL of Ni-NTA resin (Bio-Rad) in a gravity flow column (Bio-Rad). The column was washed three times with lysis buffer containing 10 mM imidazole (80 mL each), the remaining bound protein was eluted with lysis buffer containing 300 mM imidazole. Ni-NTA elution fractions containing the desired protein were combined, then concentrated buffer exchanged into 50 mM sodium phosphate (pH 7.0) 200 mM NaCl, 5 mM EDTA using an Amicon Ultra-15 centrifugal filter unit (30 kDa MWCO) (Merck Millipore). The NaCl concentration was then increase to 600 mM by adding 5 M NaCl. The protein was incubated at room temperature for 3 days to allow capsid formation. The protein was then purified through a SEC column (superpose 6 GL increase 16/600, GE

Healthcare) that had been pre-equilibrated in 50 mM sodium phosphate (pH 7.0) 150 mM NaCl, 1 mM EDTA, 1 mM TCEP-HCl. Fractions containing desired protein were combined, concentrated using an Amicon Ultra-15 centrifugal filter unit (30 kDa MWCO) (Merck Millipore) then stored at 20 °C.

***Oa*AEP1.** Recombinant expression of OaAEP1 in *E. coli*, BL21(DE3), was performed following the protocol reported previously.[2] The cells were grown at 37 °C in LB medium until the OD_{600} reached 0.6, at which point protein production was induced by adding IPTG (0.4 mM). After culturing at 16 °C for 18 h, cells were harvested by centrifugation at 5000 g and 4 °C for 20 min. Cell pellets were stored at -20 °C until purification.

The cell pellet from a 1 L culture was suspended in a lysis buffer containing 50 mM Tris-HCl (pH 7.4) 150 mM NaCl, 0.1 mg/mL lysozyme, DNase I, RNase A and PMSF. After lysis by sonication, and clearance by centrifugation (27000g, 4 °C, 15 min), the supernatant was loaded onto 4 mL of Ni-NTA resin (Bio-Rad) in a gravity flow column (Bio-Rad). The column was washed with lysis buffer containing 10 mM and 20 mM imidazole (80 mL each), the remaining bound protein was eluted with lysis buffer containing 250 mM imidazole. Ni-NTA elution fractions containing the protein were diluted and further purified by ion-exchange chromatography using 25 ml of Q-sepharose (GE Healthcare). Over 4 column volumes, bound proteins were eluted using a continuous salt gradient of 0–50% of buffer B (20 mM Bis-Tris propane (pH 7.0), 2 M NaCl). Finally, the protein was purified through a SEC column (superdex 200, GE Healthcare) that had been pre-equilibrated in 20 mM sodium phosphate (pH 7.4), 300 mM NaCl. Fractions containing desired protein were combined, concentrated using an Amicon Ultra-15 centrifugal filter unit (10 kDa MWCO) (Merck Millipore) then stored at -80 °C until activation.

OaAEP1 Activation. To activate OaAEP1, the concentrated protein stock was diluted with 50 mM sodium acetate (pH 4.0), 50 mM NaCl, 1 mM EDTA, 0.5 mM TCEP-HCl buffer to 1.5 µM. Then, the activation mixture was dialysed at 20 °C (sodium acetate buffer as described above). After 16 h, protein concentration was measure by nanodrop. The buffer of the activated protein solution was then exchanged by dialysis at 4 °C into the desired reaction buffer. Protein precipitation during activation allowed removal of the contaminating proteins by centrifugation (4000g, 4 °C, 20 min). The mature AEP can be further purified by ion exchange chromatography according to protocol reported previously.[3]

Peptide cyclization kinetic assay. Purified model peptide was synthesized by SPPS. Cyclization assays were performed in 50 µL reaction mixtures containing either 50 mM NaOAc (pH 4.5 – 5.5, 50 mM NaCl, 1 mM EDTA), or 50 mM MES (pH 5.5 – 6.0, 50 mM NaCl, 1 mM EDTA), or 20 mM Na_2HPO_4 (pH 6.0 – 7.4, 100 mM NaCl, 1 mM EDTA), supplemented with the AEP (2 to 20 nM) and peptide substrate (10 to 2000 µM). Each reaction was performed in triplicate at 20 °C and quenched after 1 h by adding 5 µL of 1 M HCl solution. The quenched reaction mixtures were analyzed by LCMS (method above). Reaction velocities were calculated by converting the HPLC peak areas of the 210 nm UV chromatogram corresponding to the remaining linear precursors and the cyclized products into concentrations. The identity of each HPLC peak was confirmed by MS. The Michaelis-Menten

curve and the kinetic parameters (k_{cat} and K_M) were estimated by curve fitting using Sigma Plot (Systat Software).

OaAEP1 substrate scope assay. Substrates peptides were either purchased from Genscript or synthesized by SPPS. Peptide ligation assays were performed in 50 µL reaction mixtures containing either 50 mM NaOAc (pH 5.0), 50 mM NaCl, 1 mM EDTA, OaAEP1-C247A (50 nM), peptide 1 (GLGGIR, 250 µM), peptide 2 (CFRANXL or CFRANGX, 50 µM). Each reaction was performed at 20 °C and quenched after 1 h by adding 5 µl of 1 M HCl solution. The quenched reaction mixtures were analyzed by LCMS (method above). Reaction yields were calculated by converting the HPLC peak areas of the 210 nm UV chromatogram corresponding to the desired peptide products into concentrations. The identity of each HPLC peak was confirmed by MS.

Reaction kinetics of N-terminal cysteine peptide coupling to 2-formylphenyl boronic acid

Peptide CFRANGL (10 µM) and 2-formylphenyl boronic acid (10 µM) were dissolved in either 50 mM NaOAc (pH 5.0) or 20 mM Na_2HPO_4 (pH 6.0 or 7.0) buffer with 100 mM NaCl, 1 mM EDTA. The reaction progress was monitored with Shimadzu UV/Vis at 20 °C for 20 min at 5 s intervals. Reaction yields and kinetics were calculated by absorbance at 254 nm.[4] Reaction was performed in triplicate.

Peptide ligation assay

Substrates peptides were synthesized by SPPS. In 50 µL, peptide 1, LFRANCLK (200 – 400 µM), peptide 2, GLGGIR (200 – 480 µM), 2-formylphenyl boronic acid (200 – 800 µM) and OaAEP1-C247A (0.2 – 0.6 µM) were dissolved in buffer (pH 4.5 - 5.0, 50 mM NaOAc, 50 mM NaCl, 1 mM EDTA and 0.5 mM TCEP, pH 5.2 – 6.5 50 mM MES buffer with 50 mM NaCl, 1 mM EDTA and 0.5 mM TCEP). The reactions were incubated at either 4 °C, 20 °C or 37 °C for up to 18 hours, then quenched with with 5 µL of 1 M HCl. As a control, the reaction was carried out in 50 mM MES (pH 5.7) at 20 °C with LFRANCLK (300 µM), GLGGIR (360 µM), 2-formylphenyl boronic acid (600 µM) and OaAEP1-C247A (0.3 µM). All reactions were performed in triplicate. Quenched reaction mixtures were analyzed by LCMS and analytical HPLC. The yields were calculated by converting the HPLC peak areas of the 210 nm UV chromatogram corresponding to the desired ligated products into concentrations. The identity of each HPLC peak was confirmed by MS.

Protein ligation assay

For protein conjugation with biotin labelled peptide, in 100 µL, substrate protein (100 µM), biotin peptide (200 µM), 2-formylphenyl boronic acid (200 µM) and OaAEP1-C247A (0.25 µM) were added to 50 mM MES buffer (pH 6.0), 50 mM NaCl, 1 mM EDTA, 0.5 mM TCEP buffer. The reactions were incubated at 20 °C for 16 hours, then quenched with 10 µL of 1 M HCl. For protein conjugation of AaLS13 with biotin labelled peptide, in 100 µL, substrate protein (100 µM), biotin peptide (200 µM), 2-formylphenyl boronic acid (200 µM) and OaAEP1 (0.5 µM) were added to 50 mM NaP_i buffer (pH 7.0), 150 mM NaCl, 1 mM EDTA, 0.5 mM TCEP buffer. The reactions were incubated at 20 °C for 18 hours, then quenched with 10 µL of 1 M HCl. Quenched reaction mixtures were analyzed by Synapt UPLC-MS. The mass spectrum was taken as the average across the whole chromatographic peak and total ion count ratio

between the protein starting material and the desired biotin labelled protein was used to estimate the reaction yield.

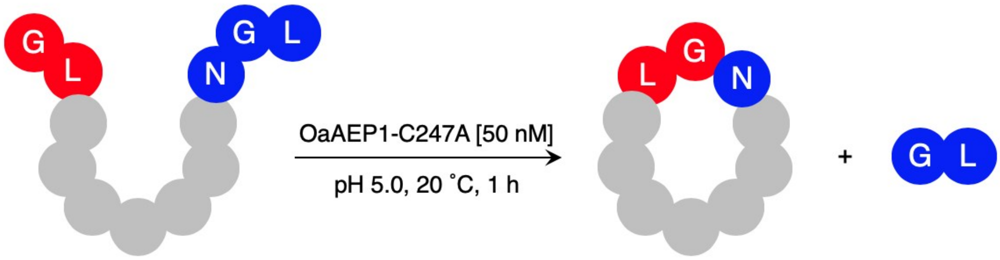

OaAEP1-C247A	Yield [%]
Crude[a]	52
Purified[b]	68

[a] Activated in 50 mM NaOAc (pH 4.0) with 50 mM NaCl, 1 mM EDTA, 0.5 mM TCEP at 20 °C for 16 h

[b] Activated as [a], then purified by SP Sepharose using 50 mM NaOAc (pH 4.0) and 50 mM NaOAc (pH 4.0), 1 M NaCl

Peptide sequence: GLPVSTRPVATRNGL

Figure S1. Peptide cyclization activity of “crude” *Oa*AEP1-C247A after acid activation compared to activated enzyme that has been “purified” by ion exchange chromatography

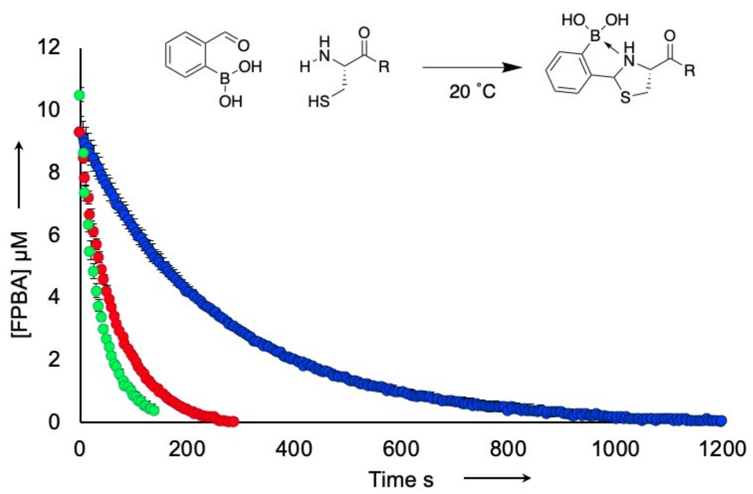

pH	k_2 [M^{-1} s^{-1}]	$T_{1/2}$ [s]
5.0	733 ± 58	124 ± 14
6.0	2700 ± 100	37 ± 1
7.0	5967 ± 306	12 ± 3

Figure S2. Kinetics of N-terminal cysteine peptide coupling to 2-formylphenyl boronic acid (FPBA). Peptide sequence: CFRANGL

GLPVSTKPVATRNGL → c-GLPVSTKPVATRN

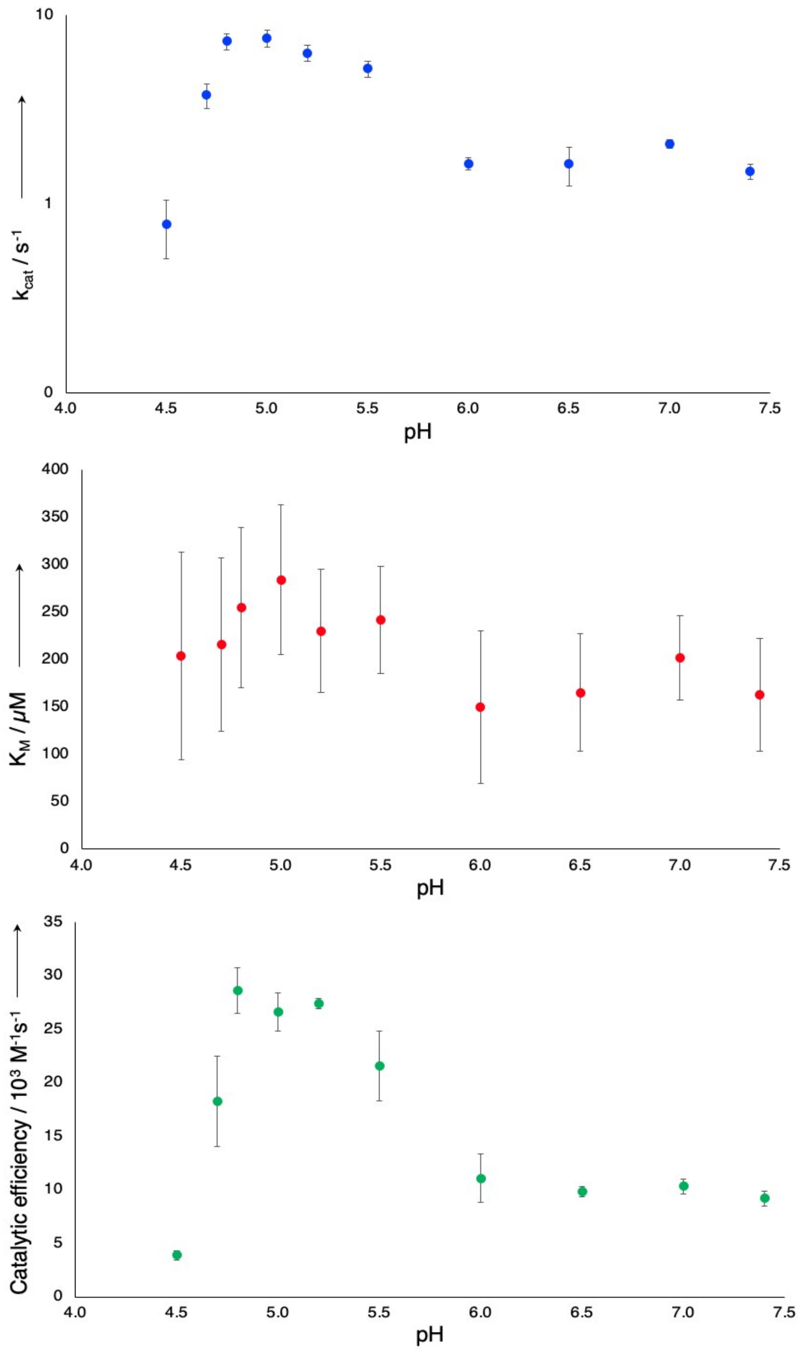

Figure S3. the pH dependence of the OaAEP1-catalyzed peptide cyclisation. The k_{cat}, K_M and catalytic efficiency ($k_{cat}/_{KM}$) are shown in blue, red and green, respectively.

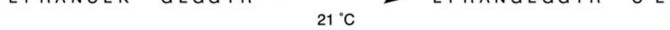

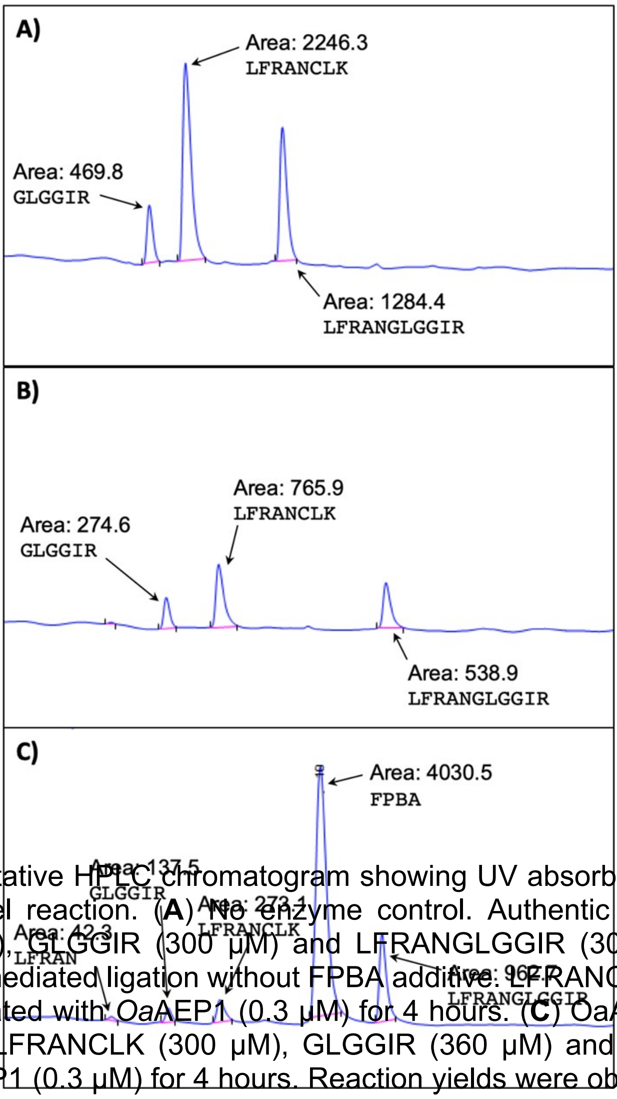

Figure S4. Representative HPLC chromatogram showing UV absorbance at 210 nm for the peptide ligation model reaction. (**A**) No enzyme control. Authentic standards of peptides LFRANCLK (300 µM), GLGGIR (300 µM) and LFRANGLGGIR (300 µM) synthesized by SPPS. (**B**) OaAEP1 mediated ligation without FPBA additive. LFRANCLK (300 µM), GLGGIR (360 µM) were incubated with *Oa*AEP1 (0.3 µM) for 4 hours. (**C**) OaAEP1 mediated ligation with FPBA additive. LFRANCLK (300 µM), GLGGIR (360 µM) and FPBA (600 µM) were incubated with *Oa*AEP1 (0.3 µM) for 4 hours. Reaction yields were obtained by integration of the UV absorbance peak area at 210 nm corresponding to the product peptide LFRANGLGGIR in comparison with a calibration curve.

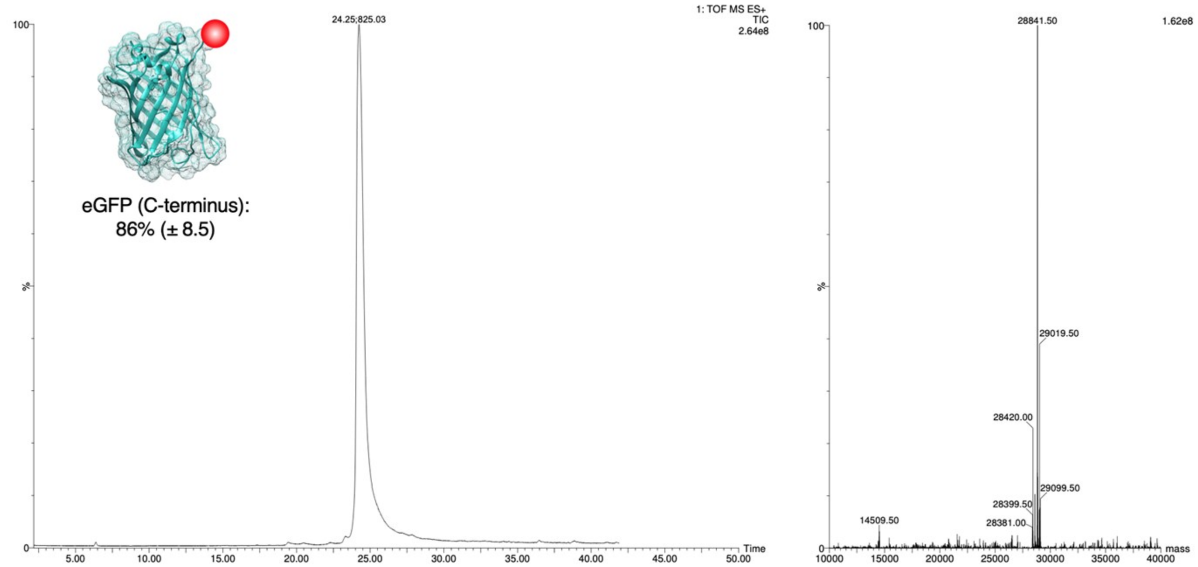

	Calculated MW	Amino acid sequence
**Starting Material (SM)**	28440.15<br>28420.15 (GFP)	MHHHHHHMVSKGEELFTGVVPILVELDGDVNGHKFSVSGEGEGD<br>ATYGKLTLKFICTTGKLPVPWPTLVTTLTYGVQCFSRYPDHMKQ<br>HDFFKSAMPEGYVQERTIFFKDDGNYKTRAEVKFEGDTLVNRIE<br>LKGIDFKEDGNILGHKLEYNYNSHNVYIMADKQKNGIKVNFKIR<br>HNIEDGSVQLADHYQQNTPIGDGPVLLPDNHYLSTQSALSKDPN<br>EKRDHMVLLEFVTAAGITLGMAELYKGGSGNCL
**Desired Product (P)**	28862.65<br>28842.65 (GFP)	MHHHHHHMVSKGEELFTGVVPILVELDGDVNGHKFSVSGEGEGD<br>ATYGKLTLKFICTTGKLPVPWPTLVTTLTYGVQCFSRYPDHMKQ<br>HDFFKSAMPEGYVQERTIFFKDDGNYKTRAEVKFEGDTLVNRIE<br>LKGIDFKEDGNILGHKLEYNYNSHNVYIMADKQKNGIKVNFKIR<br>HNIEDGSVQLADHYQQNTPIGDGPVLLPDNHYLSTQSALSKDPN<br>EKRDHMVLLEFVTAAGITLGMAELYKGGSGNGLGGZ
**Hydrolyzed side Product (SP)**	28223.85<br>28203.85 (GFP)	MHHHHHHMVSKGEELFTGVVPILVELDGDVNGHKFSVSGEGEGD<br>ATYGKLTLKFICTTGKLPVPWPTLVTTLTYGVQCFSRYPDHMKQ<br>HDFFKSAMPEGYVQERTIFFKDDGNYKTRAEVKFEGDTLVNRIE<br>LKGIDFKEDGNILGHKLEYNYNSHNVYIMADKQKNGIKVNFKIR<br>HNIEDGSVQLADHYQQNTPIGDGPVLLPDNHYLSTQSALSKDPN<br>EKRDHMVLLEFVTAAGITLGMAELYKGGSGN

Figure S5 Bioconjugation of biotin labeled peptide to the C-terminus of eGFP. Chromatogram and mass spectra of the UPLC-MS analysis. Label peptide sequence: GLGGZ (Z = biotinylated lysine).

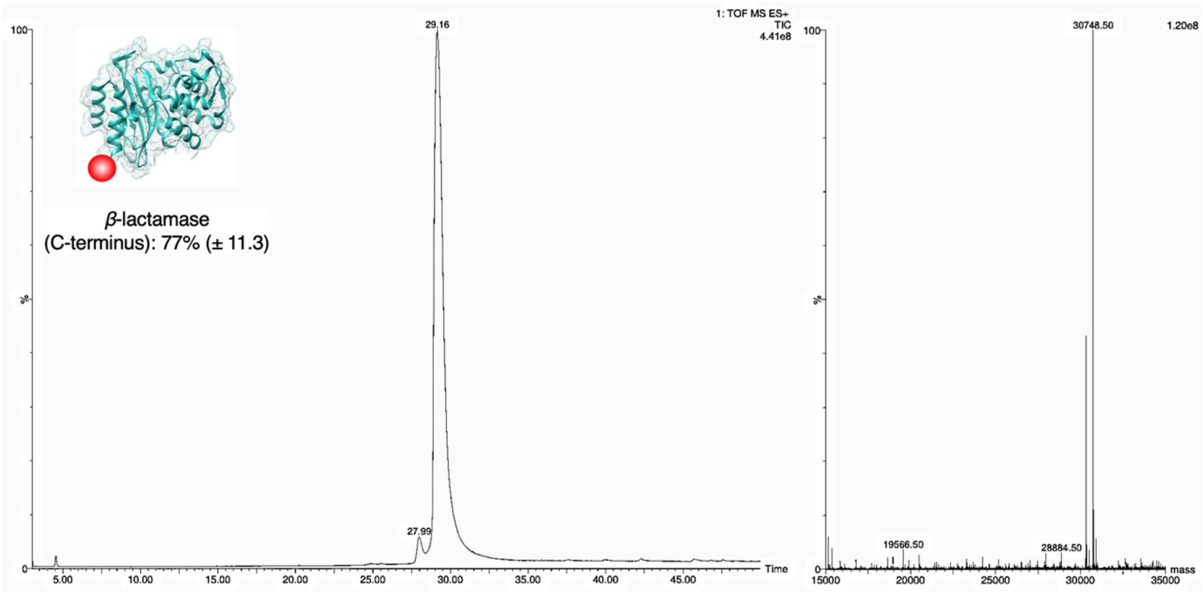

	Calculated MW	Amino acid sequence
**Starting Material (SM)**	30327.05	MHHHHHHHMGADLADRFAELERRYDARLGVYVPATGTTAAIEYR ADERFAFCSTFKAPLVAAVLHQNPLTHLDKLITYTSDDIRSISP VAQQHVQTGMTIGQLCDAAIRYSDGTAANLLLADLGGPGGGTAA FTGYLRSLGDTVSRLDAEEPELNRDPPGDERDTTTPHAIALVLQ QLVLGNALPPDKRALLTDWMARNTTGAKRIRAGFPADWKVIDKT GTGDYGRANDIAVVWSPTGVPYVVAVMSDRAGGGYDAEPREALL AEAATCVAGVLAGSGGSGNCL
**Desired Product (P)**	30749.56	MHHHHHHHMGADLADRFAELERRYDARLGVYVPATGTTAAIEYR ADERFAFCSTFKAPLVAAVLHQNPLTHLDKLITYTSDDIRSISP VAQQHVQTGMTIGQLCDAAIRYSDGTAANLLLADLGGPGGGTAA FTGYLRSLGDTVSRLDAEEPELNRDPPGDERDTTTPHAIALVLQ QLVLGNALPPDKRALLTDWMARNTTGAKRIRAGFPADWKVIDKT GTGDYGRANDIAVVWSPTGVPYVVAVMSDRAGGGYDAEPREALL AEAATCVAGVLAGSGGSGNGLGGZ
**Hydrolyzed side Product (SP)**	30110.76	MHHHHHHHMGADLADRFAELERRYDARLGVYVPATGTTAAIEYR ADERFAFCSTFKAPLVAAVLHQNPLTHLDKLITYTSDDIRSISP VAQQHVQTGMTIGQLCDAAIRYSDGTAANLLLADLGGPGGGTAA FTGYLRSLGDTVSRLDAEEPELNRDPPGDERDTTTPHAIALVLQ QLVLGNALPPDKRALLTDWMARNTTGAKRIRAGFPADWKVIDKT GTGDYGRANDIAVVWSPTGVPYVVAVMSDRAGGGYDAEPREALL AEAATCVAGVLAGSGGSGN

Figure S6 Bioconjugation of biotin labeled peptide to the C-terminus of BlaC. Chromatogram and mass spectra of the UPLC-MS analysis. Label peptide sequence: GLGGZ (Z = biotinylated lysine).

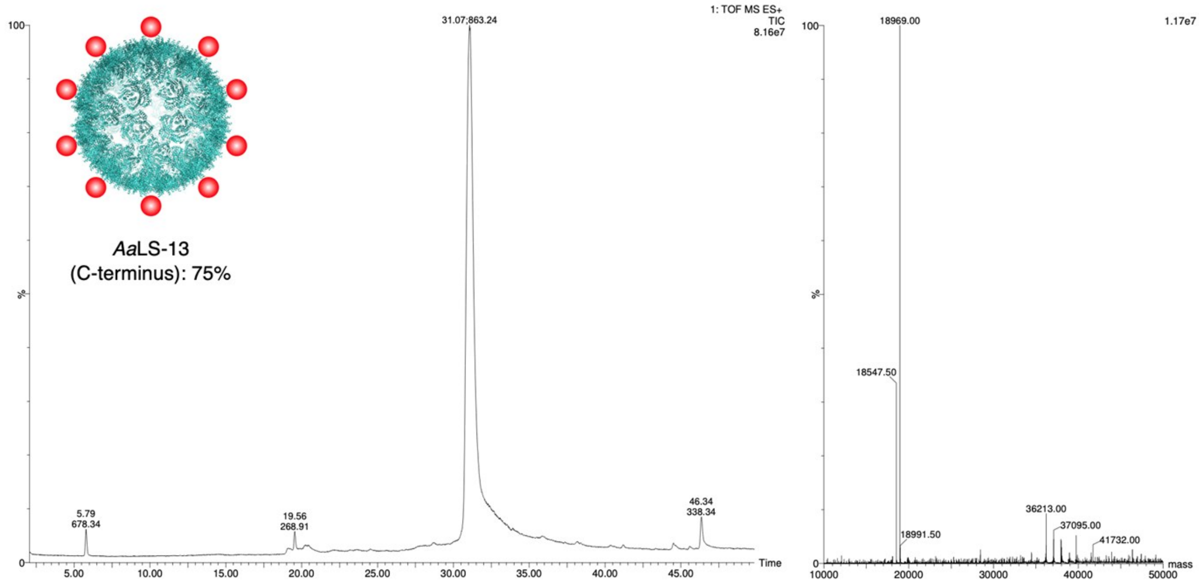

	Calculated MW	Amino acid sequence
**Starting Material (SM)**	18547.97	MHHHHHHMEIYEGKLTAEGLRFGIVASRFNHALVGRLVEGAIDC IVRHGGREEDITLVCVPGSWEIPVAAGELARKEDIDAVIAIGVL IEGAEPHFDYIASEVSKGLANLSLELRKPISFGDITDDELEEAI ECAGTEHGNKGWEAALSAIEMANLFKSLRLEGSGGSGNCL
**Desired Product (P)**	18970.47	MHHHHHHMEIYEGKLTAEGLRFGIVASRFNHALVGRLVEGAIDC IVRHGGREEDITLVCVPGSWEIPVAAGELARKEDIDAVIAIGVL IEGAEPHFDYIASEVSKGLANLSLELRKPISFGDITDDELEEAI ECAGTEHGNKGWEAALSAIEMANLFKSLRLEGSGGSGNGLGGZ
**Hydrolyzed side Product (SP)**	18331.67	MHHHHHHMEIYEGKLTAEGLRFGIVASRFNHALVGRLVEGAIDC IVRHGGREEDITLVCVPGSWEIPVAAGELARKEDIDAVIAIGVL IEGAEPHFDYIASEVSKGLANLSLELRKPISFGDITDDELEEAI ECAGTEHGNKGWEAALSAIEMANLFKSLRLEGSGGSGN

Figure S7 Bioconjugation of biotin labeled peptide to the C-terminus of AaLS13. Chromatogram and mass spectra of the UPLC-MS analysis. Label peptide sequence: GLGGZ (Z = biotinylated lysine).

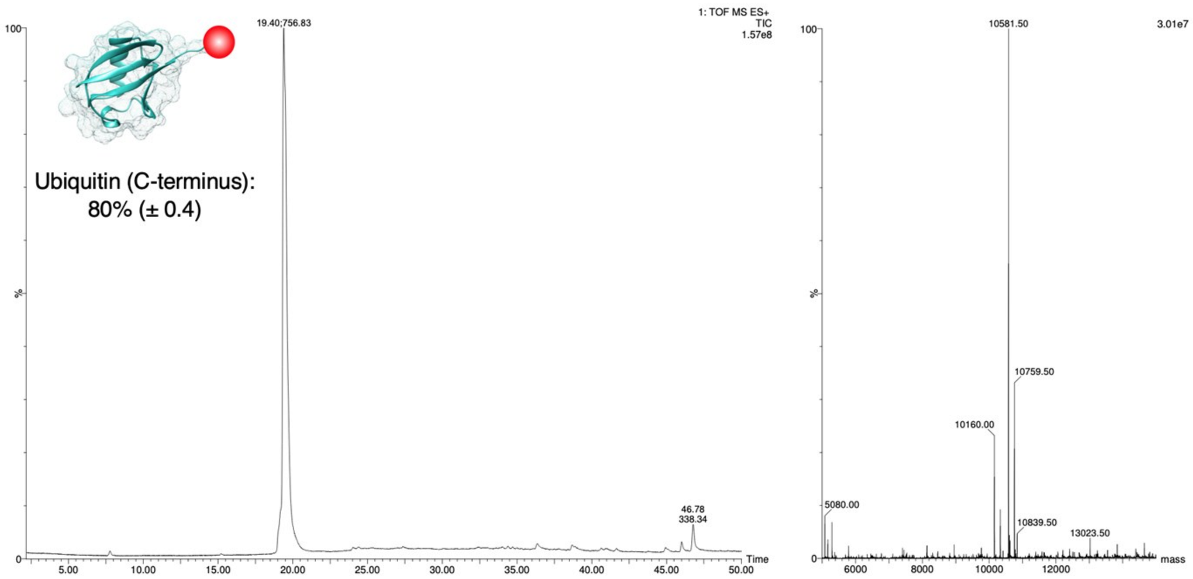

	Calculated MW	Amino acid sequence
**Starting Material (SM)**	10160.52	MHHHHHHQIFVKTLTGKTITLEVEPSDTIENVKAKIQDKEGIPPDQQRLIFAGKQLEDGRTLSDYNIQKESTLHLVLRLRGGPSGGSGNCL
**Desired Product (P)**	10583.02	MHHHHHHQIFVKTLTGKTITLEVEPSDTIENVKAKIQDKEGIPPDQQRLIFAGKQLEDGRTLSDYNIQKESTLHLVLRLRGGPSGGSGNGLGGZ
**Hydrolyzed side Product (SP)**	9944.22	MHHHHHHQIFVKTLTGKTITLEVEPSDTIENVKAKIQDKEGIPPDQQRLIFAGKQLEDGRTLSDYNIQKESTLHLVLRLRGGPSGGSGN

Figure S8 Bioconjugation of biotin labeled peptide to the C-terminus of ubiquitin. Chromatogram and mass spectra of the UPLC-MS analysis. Label peptide sequence: GLGGZ (Z = biotinylated lysine).

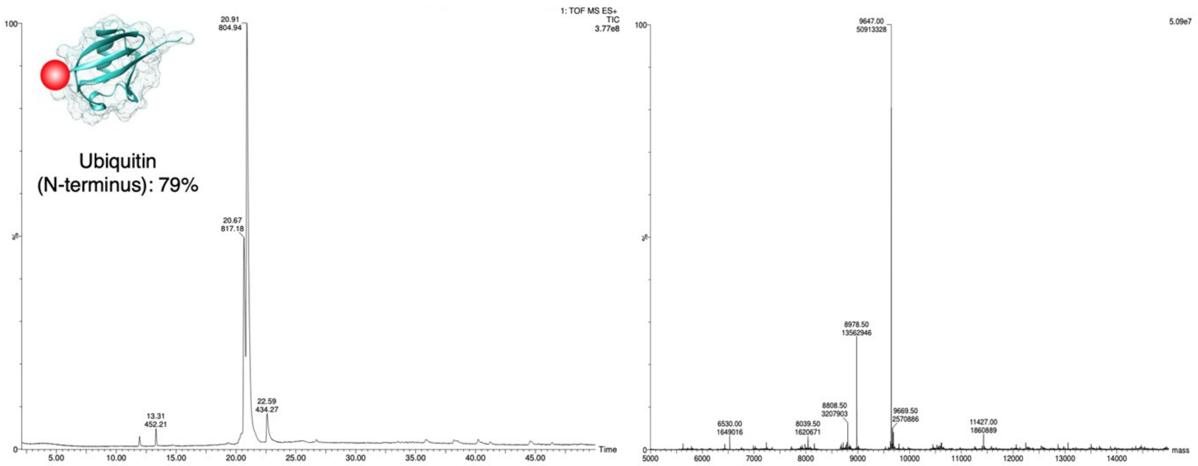

	Calculated MW	Amino acid sequence
**Starting Material (SM)**	8979.20	GLSGSGSQIFVKTLTGKTITLEVEPSDTIENVKAKIQDKEGIPP DQQRLIFAGKQLEDGRTLSDYNIQKESTLHLVLRLRGG
**Desired Product (P)**	9647.99	XTRNGLSGSGSQIFVKTLTGKTITLEVEPSDTIENVKAKIQDKE GIPPDQQRLIFAGKQLEDGRTLSDYNIQKESTLHLVLRLRGG

Figure S9 Bioconjugation of biotin labeled peptide to the N-terminus of ubiquitin. Chromatogram and mass spectra of the UPLC-MS analysis. The two LC peaks correspond to starting material and products. Label peptide sequence: XTRNCL (X = biotinylated alanine).

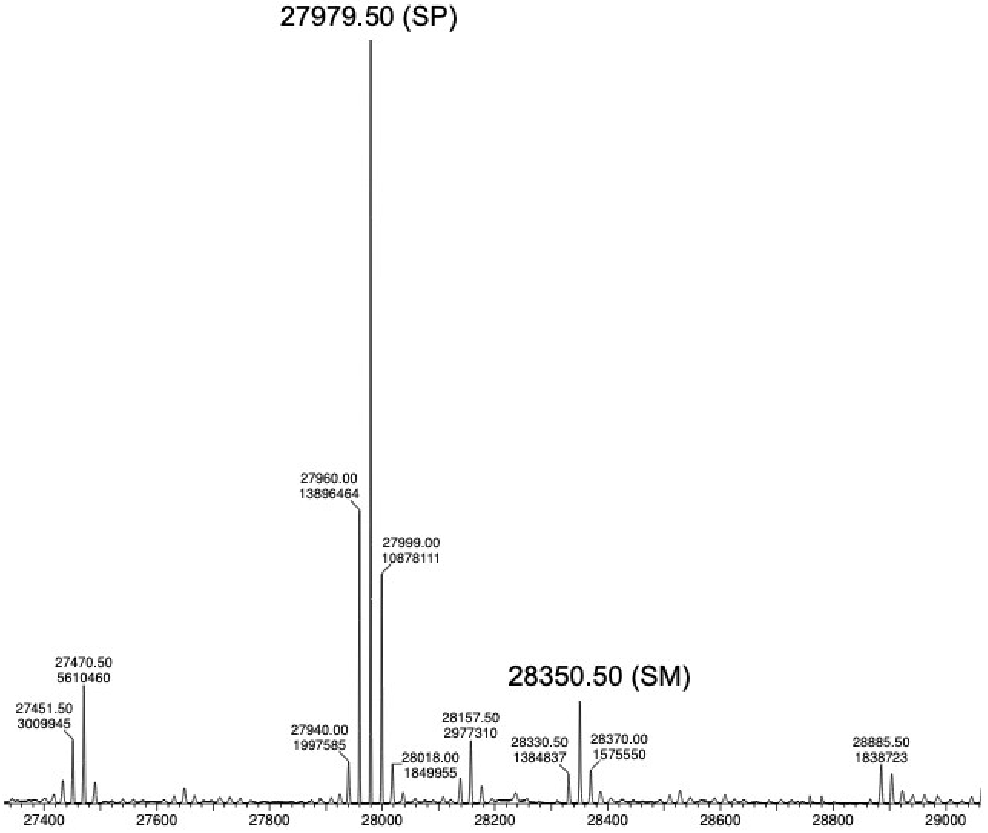

	Calculated MW	Amino acid sequence
**Starting Material (SM)**	28484.16<br>28352.96 (-Met)	MHHHHHHMVSKGEELFTGVVPILVELDGDVNGHKFSVSGEGEGD<br>ATYGKLTLKFICTTGKLPVPWPTLVTTLTYGVQCFSRYPDHMKQ<br>HDFFKSAMPEGYVQERTIFFKDDGNYKTRAEVKFEGDTLVNRIE<br>LKGIDFKEDGNILGHKLEYNYNSHNVYIMADKQKNGIKVNFKIR<br>HNIEDGSVQLADHYQQNTPIGDGPVLLPDNHYLSTQSALSKDPN<br>EKRDHMVLLEFVTAAGITLGMDELYKGGSGNCL
**Desired Product (P)**	28906.66<br>28886.66 (GFP)	MHHHHHHMVSKGEELFTGVVPILVELDGDVNGHKFSVSGEGEGD<br>ATYGKLTLKFICTTGKLPVPWPTLVTTLTYGVQCFSRYPDHMKQ<br>HDFFKSAMPEGYVQERTIFFKDDGNYKTRAEVKFEGDTLVNRIE<br>LKGIDFKEDGNILGHKLEYNYNSHNVYIMADKQKNGIKVNFKIR<br>HNIEDGSVQLADHYQQNTPIGDGPVLLPDNHYLSTQSALSKDPN<br>EKRDHMVLLEFVTAAGITLGMDELYKGGSGNGLGGZ
**Side Product (SP)**	28000.69<br>27980.69 (GFP)	MHHHHHHMVSKGEELFTGVVPILVELDGDVNGHKFSVSGEGEGD<br>ATYGKLTLKFICTTGKLPVPWPTLVTTLTYGVQCFSRYPDHMKQ<br>HDFFKSAMPEGYVQERTIFFKDDGNYKTRAEVKFEGDTLVNRIE<br>LKGIDFKEDGNILGHKLEYNYNSHNVYIMADKQKNGIKVNFKIR<br>HNIEDGSVQLADHYQQNTPIGDGPVLLPDNHYLSTQSALSKDPN<br>EKRDHMVLLEFVTAAGITLGMDGLGGZ

Figure S10. Bioconjugation of biotin labeled peptide to the C-terminus of eGFP. OaAEP1 mediates the ligation reaction at an alternative recognition site within the native protein backbone. Label peptide sequence: GLGGZ (Z = biotinylated lysine)

Table S1. Kinetic parameters of OaAEP1-C247A obtained from acid activation of the zymogen and direct expression of the catalytic core domain

Entry	Source	Activation	k_{cat} (s^{-1})	K_M (μM)	k_{cat} / K_M ($M^{-1}s^{-1}$)
1[a]*	Literature[5]	Acid	1.4 ± 0.1	3.6 ± 1.1	3.9 x 10^5
2[b]**	This work+	Acid	7.5 ± 0.8	284 ± 79	2.7 x 10^4
3[c]**	Literature[2]	Acid	13.9 ± 1.1	407 ± 49	3.4 x 10^4
4[b]**	This work++	Acid	1.6 ± 0.1	150 ± 80	1.1 x 10^4
5[b]**	This work++	N/A	1.7± 0.2	201 ± 59	0.8 x 10^4

[a] Assay at 25 °C. Data from ref ([5]). [b] Assay at 20 °C. [c] Assay at 37 °C. Data obtained from ref ([2]).

+ pH 5.0
++ pH 6.0
* Substrate peptide sequence: Abz-RNGLG-Y($3NO_2$)
** Substrate peptide sequence: GLPVSTKPVATRNGL

The kinetic parameters of OaAEP1-C247A were previously reported with two different methods, which employ different substrate, buffers, pH and temperature. Nevertheless, the kinetic parameters obtained from this study lies within the range of the values reported in the literature (for both k_{cat} and K_M). We therefore concluded that the new, simplified enzyme construct showed no significant difference in activity when compared to the OaAEP1-C247A obtained *via* acid activation. In this work, both acid-activated OaAEP1-C247A and the new construct obtained by direct expression were used for both kinetic characterization and protein labeling.

Table S2 Optimization of model peptide ligation, where the ligation of LFRANCLK to GLGGIR is catalysed by OaAEP1-C247A in the presence or absence of FPBA

Entry	Temperature °C	pH	Time h	[OaAEP1] μM	[FPBA] μM	[LFRANCLK] μM	[GLGGIR] μM	Yield %	Hydrolysis %
1	4	4.5[a]	2	0.4	0	400	480	27	3
2	4	4.5[a]	2	0.4	800	400	480	3	1
3	4	4.5[a]	3	0.4	0	400	480	33	8
4	4	4.5[a]	3	0.4	800	400	480	8	1
5	4	5.0[a]	1	0.1	400	200	200	23	1
6	4	5.0[a]	1	0.1	400	200	240	24	1
7	4	5.0[a]	1	0.1	400	200	400	34	1
8	4	5.0[a]	1	0.1	600	200	200	17	0
9	4	5.0[a]	1	0.2	200	200	200	25	13
10	4	5.0[a]	1	0.2	400	200	200	49	3
11	4	5.0[a]	1	0.4	400	200	200	55	11
12	4	5.0[a]	1	0.4	400	400	400	47	12
13	4	5.0[a]	1	0.6	400	200	200	46	50
14	4	5.0[a]	2	0.1	400	200	200	35	3
15	4	5.0[a]	2	0.1	400	200	240	37	1
16	4	5.0[a]	2	0.1	400	200	400	42	1
17	4	5.0[a]	2	0.1	600	200	200	31	1
18	4	5.0[a]	2	0.2	200	200	200	51	23
19	4	5.0[a]	2	0.2	400	200	200	64	9
20	4	5.0[a]	2	0.4	400	200	200	65	21
21	4	5.0[a]	2	0.4	400	400	400	66	25
22	4	5.0[a]	2	0.6	400	200	200	33	66
23	4	5.0[a]	3	0.1	400	200	200	43	4
24	4	5.0[a]	3	0.1	400	200	240	46	2
25	4	5.0[a]	3	0.1	400	200	400	49	1
26	4	5.0[a]	3	0.1	600	200	200	42	1
27	4	5.0[a]	3	0.2	400	200	200	60	13
28	4	5.0[a]	3	0.4	400	200	200	64	31
29	4	5.0[a]	3	0.6	400	200	200	27	72
30	4	5.0[a]	4	0.1	400	200	200	47	5
31	4	5.0[a]	4	0.1	400	200	240	50	2
32	4	5.0[a]	4	0.1	400	200	400	53	1
33	4	5.0[a]	4	0.1	600	200	200	44	1
34	4	5.0[a]	4	0.2	400	200	200	64	20
35	4	5.0[a]	4	0.4	400	200	200	57	38
36	4	5.0[a]	4	0.6	400	200	200	13	87
37	4	5.0[a]	18	0.2	200	200	200	43	35
38	20	4.5[a]	2	0.2	0	200	200	35	8
39	20	4.5[a]	2	0.2	200	200	200	49	9

40	20	4.5[a]	2	0.4	0	400	480	39	14
41	20	4.5[a]	2	0.4	800	400	480	58	17
42	20	4.5[a]	3	0.4	0	400	480	38	25
43	20	4.5[a]	3	0.4	800	400	480	69	19
44	20	4.7[a]	2	0.2	0	200	200	36	8
45	20	4.7[a]	2	0.2	200	200	200	49	5
46	20	5.0[a]	0.2	0.2	200	200	200	15	14
47	20	5.0[a]	0.5	0.2	200	200	200	38	19
48	20	5.0[a]	1	0.2	200	200	200	48	20
49	20	5.0[a]	1	0.4	400	400	400	62	18
50	20	5.0[a]	2	0.2	0	200	200	33	21
51	20	5.0[a]	2	0.2	200	200	200	58	32
52	20	5.0[a]	2	0.2	400	200	200	67	27
53	20	5.0[a]	2	0.3	0	300	360	42	7
54	20	5.0[a]	2	0.3	600	300	360	75	9
55	20	5.0[a]	2	0.4	400	400	400	70	23
56	20	5.0[a]	3	0.3	0	300	360	53	13
57	20	5.0[a]	3	0.3	600	300	360	89	10
58	20	5.0[a]	18	0.4	400	400	400	25	73
59	20	5.0[a]	18	0.2	200	200	200	18	78
60	20	5.2[a]	2	0.3	0	300	360	52	3
61	20	5.2[a]	2	0.3	600	300	360	73	4
62	20	5.2[a]	3	0.3	0	300	360	54	3
63	20	5.2[a]	3	0.3	600	300	360	95	3
64	20	5.5[a]	2	0.3	0	300	360	48	1
65	20	5.5[a]	2	0.3	600	300	360	69	0
66	20	5.5[a]	3	0.3	0	300	360	49	2
67	20	5.5[a]	3	0.3	600	300	360	85	1
68	20	5.5[a]	4	0.3	0	300	360	48	3
69	20	5.5[a]	4	0.3	600	300	360	90	5
70	20	5.7[b]	2	0.3	0	300	360	51	1
71	20	5.7[b]	2	0.3	600	300	360	57	0
72	20	5.7[b]	3	0.3	0	300	360	51	1
73	20	5.7[b]	3	0.3	600	300	360	78	2
74	20	5.7[b]	4	0.3	0	300	360	50	2
75	20	5.7[b]	4	0.3	600	300	360	94	3
76	20	5.9[b]	2	0.3	0	300	360	58	0
77	20	5.9[b]	2	0.3	600	300	360	28	0
78	20	5.9[b]	3	0.3	0	300	360	56	1
79	20	5.9[b]	3	0.3	600	300	360	66	1
80	20	6.0[b]	2	0.3	0	300	360	53	0
81	20	6.0[b]	2	0.3	600	300	360	32	0
82	20	6.0[b]	3	0.3	0	300	360	54	0

83	20	6.0[b]	3	0.3	600	300	360	55	0
84	20	6.0[b]	4	0.3	0	300	360	56	1
85	20	6.0[b]	4	0.3	600	300	360	77	0
86	20	6.5[b]	2	0.3	0	300	360	19	0
87	20	6.5[b]	2	0.3	600	300	360	15	0
88	20	6.5[b]	3	0.3	0	300	360	57	0
89	20	6.5[b]	3	0.3	600	300	360	20	0
90	20	6.5[b]	4	0.3	0	300	360	55	0
91	20	6.5[b]	4	0.3	600	300	360	22	1
92	37	5.0[a]	0.2	0.2	200	200	200	17	13
93	37	5.0[a]	0.5	0.2	200	200	200	44	23
94	37	5.0[a]	1	0.2	200	200	200	48	44
95	37	5.0[a]	2	0.2	0	200	200	30	51
96	37	5.0[a]	2	0.2	200	200	200	49	43
97	37	6.5[b]	2	0.3	0	300	360	33	0
98	37	6.5[b]	2	0.3	600	300	360	53	0
99	37	6.5[b]	3	0.3	0	300	360	53	0
100	37	6.5[b]	3	0.3	600	300	360	82	0
101	37	6.5[b]	4	0.3	0	300	360	55	0
102	37	6.5[b]	4	0.3	600	300	360	81	0

[a] 50 mM NaOAc buffer with 50 mM NaCl, 1 mM EDTA, 0.5 mM TCEP [b] 50 mM MES buffer with 50 mM NaCl, 1 mM EDTA, 0.5 mM TCEP

Table S3. C-terminus labeling of proteins with biotinylated peptide using OaAEP1-C247A in the absence of 2-formyl phenylboronic acid (FPBA)

Protein	GLGGZ (equiv.)	Yield (%)	Hydrolysis (%)
eGFP	1.0	43	4
eGFP	1.2	58	1
eGFP	1.5	64	5
eGFP	1.7	72	2
eGFP	2.0	71	5
Ubiquitin	2.0	60	6
β Lactamase	2.0	56	0

eGFP-NCL (100 μM), GLGGZ (100-200 μM), OaAEP1 (0.25 μM), 2-FPBA (200 μM) in 50 mM MES buffer (pH 6.0), 50 mM NaCl, 1 mM EDTA, at 20 °C

Table S4. Amino acid sequence of protein substrates used for bioconjugation. Underlined sequence corresponds to the OaAEP1-C247A recognition sequence. Highlighted in red are recognition sequence of OaAEP1 found in the native peptide sequence. For eGFP a site directed mutagenesis was carried out to prevent the undesired truncation during the OaAEP1 reaction. This side reaction was not observed in AaLS13 despite the presence of the same recognition sequence.

**Protein**	**Sequence**
His_6 TEV-tagged Ubiquitin for N-terminal labeling	MGHHHHHHGENLYFQGLSGSGSQIFVKTLTGKTITLEVEPSDTIENVKAKIQDK<br>EGIPPDQQRLIFAGKQLEDGRTLSDYNIQKESTLHLVLRLRGG
His_6 tagged Ubiquitin for C-terminal labeling	MHHHHHHQIFVKTLTGKTITLEVEPSDTIENVKAKIQDKEGIPPDQQRLIFAGK<br>QLEDGRTLSDYNIQKESTLHLVLRLRGGPSGGSGNCL
His_6 tagged eGFP for C-terminal labeling (led to side product formation)	MHHHHHHMVSKGEELFTGVVPILVELDGDVNGHKFSVSGEGEGDATYGKLTLKF<br>ICTTGKLPVPWPTLVTTLTYGVQCFSRYPDHMKQHDFFKSAMPEGYVQERTIFF<br>KDDGNYKTRAEVKFEGDTLVNRIELKGIDFKEDGNILGHKLEYNYNSHNVYIMA<br>DKQKNGIKVNFKIRHNIEDGSVQLADHYQQNTPIGDGPVLLPDNHYLSTQSALS<br>KDPNEKRDHMVLLEFVTAAGITLGMDELYKGGSGNCL
His_6 tagged eGFP for C-terminal labeling with no side product observed	MHHHHHHMVSKGEELFTGVVPILVELDGDVNGHKFSVSGEGEGDATYGKLTLKF<br>ICTTGKLPVPWPTLVTTLTYGVQCFSRYPDHMKQHDFFKSAMPEGYVQERTIFF<br>KDDGNYKTRAEVKFEGDTLVNRIELKGIDFKEDGNILGHKLEYNYNSHNVYIMA<br>DKQKNGIKVNFKIRHNIEDGSVQLADHYQQNTPIGDGPVLLPDNHYLSTQSALS<br>KDPNEKRDHMVLLEFVTAAGITLGMAELYKGGSGNCL
His_6 tagged BlaC for C-terminal labeling	MHHHHHHMGADLADRFAELERRYDARLGVYVPATGTTAAIEYRADERFAFCSTF<br>KAPLVAAVLHQNPLTHLDKLITYTSDDIRSISPVAQQHVQTGMTIGQLCDAAIR<br>YSDGTAANLLLADLGGPGGGTAAFTGYLRSLGDTVSRLDAEEPELNRDPPGDER<br>DTTTPHAIALVLQQLVLGNALPPDKRALLTDWMARNTTGAKRIRAGFPADWKVI<br>DKTGTGDYGRANDIAVVWSPTGVPYVVAVMSDRAGGGYDAEPREALLAEAATCV<br>AGVLAGSGGSGNCL
His_6 tagged AaLS13 for C-terminal labeling	MHHHHHHMEIYEGKLTAEGLRFGIVASRFNHALVGRLVEGAIDCIVRHGGREED<br>ITLVCVPGSWEIPVAAGELARKEDIDAVIAIGVLIEGAEPHFDYIASEVSKGLA<br>NLSLELRKPISFGDITDDELEEAIECAGTEHGNKGWEAALSAIEMANLFKSLRL<br>EGSGGSGNCL

Reference:

(1) Azuma, Y.; Zschoche, R.; Tinzl, M.; Hilvert, D. Quantitative Packaging of Active Enzymes into a Protein Cage. *Angew. Chemie Int. Ed.* **2016**, *55* (4), 1531–1534. https://doi.org/10.1002/anie.201508414.

(2) Yang, R. L.; Wong, Y. H.; Nguyen, G. K. T.; Tam, J. P.; Lescar, J.; Wu, B. Engineering a Catalytically Efficient Recombinant Protein Ligase. *J. Am. Chem. Soc.* **2017**, *139* (15), 5351–5358. https://doi.org/10.1021/jacs.6b12637.

(3) Harris, K. S.; Guarino, R. F.; Dissanayake, R. S.; Quimbar, P.; McCorkelle, O. C.; Poon, S.; Kaas, Q.; Durek, T.; Gilding, E. K.; Jackson, M. A.; et al. A Suite of Kinetically Superior AEP Ligases Can Cyclise an Intrinsically Disordered Protein. *Sci. Rep.* **2019**, *9* (1), 10820. https://doi.org/10.1038/s41598-019-47273-7.

(4) Bandyopadhyay, A.; Cambray, S.; Gao, J. Fast and Selective Labeling of N-Terminal Cysteines at Neutral PH via Thiazolidino Boronate Formation. *Chem. Sci.* **2016**, *7* (7), 4589–4593. https://doi.org/10.1039/C6SC00172F.

(5) Rehm, F. B. H.; Harmand, T. J.; Yap, K.; Durek, T.; Craik, D. J.; Ploegh, H. L. Site-Specific Sequential Protein Labeling Catalyzed by a Single Recombinant Ligase. *J. Am. Chem. Soc.* **2019**, *141* (43), 17388–17393. https://doi.org/10.1021/jacs.9b09166.
